# Occupational modulation in the (3+1)-dimensional incommensurate structure of (2*S*,3*S*)-2-amino-3-hy­droxy-3-methyl-4-phen­oxy­butanoic acid dihydrate

**DOI:** 10.1107/S2053229624007009

**Published:** 2024-08-08

**Authors:** Kyana M. Sanders, Samantha K. Bruffy, Andrew R. Buller, Václav Petříček, Ilia A. Guzei

**Affiliations:** a Department of Chemistry, University of Wisconsin-Madison, 1101 University Ave, Madison, WI 53706, USA; b Department of Structure Analysis, Institute of Physics of the Czech Academy of Sciences, Na Slovance 2, 182 00 Prague 8, Czech Republic; University of Oxford, United Kingdom

**Keywords:** crystal structure, modulated structure, incommensurate modulation, supercell approximation, occupational/positional disorder, hy­dro­gen bonding

## Abstract

The incommensurately modulated structure of (2*S*,3*S*)-2-amino-3-hy­droxy-3-methyl-4-phen­oxy­butanoic acid dihydrate (C_11_H_15_NO_4_·2H_2_O or **I**·2H_2_O) is described in the (3+1)-dimensional superspace group *P*2_1_2_1_2_1_(0β0)000 (β = 0.357). The loss of the three-dimensional periodicity is ascribed to the occupational modulation of one positionally disordered solvent water mol­ecule, where the two positions are related by a small translation [*ca* 0.666 (9) Å] and ∼168 (5)° rotation about one of its O—H bonds, with an average 0.624 (3):0.376 (3) occupancy ratio. The occupational modulation of this mol­ecule arises due to the com­petition between the different hy­dro­gen-bonding motifs associated with each position. The structure can be very well refined in the average approximation (all satellite reflections disregarded) in the space group *P*2_1_2_1_2_1_, with the water mol­ecule refined as disordered over two positions in a 0.625 (16):0.375 (16) ratio. The refinement in the commensurate threefold supercell approximation in the space group *P*112_1_ is also of high quality, with the six corresponding water mol­ecules exhibiting three different occupancy ratios averaging 0.635:0.365.

## Abstract 2

The (3+1)-dimensional incommensurate structure of (2*S*,3*S*)-2-amino-3-hy­droxy-3-methyl-4-phen­oxy­butanoic acid dihydrate (**I**·2H_2_O), in which one of the water mol­ecules is disordered over two positions and each position exhibits occupational modulation, can be well refined in superspace, and in the average and supercell approximations. The occupational modulation of the disordered solvent water mol­ecule results from the com­petition between the different hy­dro­gen-bonding motifs associated with the two disorder positions of the water mol­ecule.

## Introduction

The implementation of enzymes in industry has advanced the synthesis of pharmaceuticals and bioactive com­pounds. Still, the use of enzymes is dwarfed in com­parison to the plethora of established organic transformations. In particular, successful examples of enzyme-catalyzed C—C bond formation reactions with simple C-nucleophiles and C-electrophiles are limited. The Buller Lab has recently characterized an l-threonine transaldolase, ObiH, which generates a high-energy carbanion inter­mediate that is shielded from protonation (Kumar *et al.*, 2021 ▸; Doyon *et al.*, 2022 ▸). Natively, the nucleophilic inter­mediate inter­cepts a phenyl­acetaldehyde electrophile enan­tio­selectively, producing the β-OH amino acid (2*S*,3*R*)-2-amino-3-hy­droxy-4-(4-nitro­phen­yl)butanoic acid, an inter­mediate in obafluorin biosynthesis (Schaffer *et al.*, 2017 ▸; Scott *et al.*, 2017 ▸). It was hypothesized that the kinetically trapped inter­mediate of ObiH may enable productive catalysis with even less reactive electrophiles, such as ketones, generating tertiary β-OH amino acid. Such tertiary alcohols are a com­mon motif in bioactive mol­ecules, but their enan­tio­selective synthesis is a long-standing challenge in both traditional synthetic chemistry and biocatalysis. Phen­oxy­pro­pan-2-one was selected as a substrate for the ObiH reaction to explore nonnative aldol addition activity to make a tertiary alcohol. Analytical reactions showed evidence of good conversion (>10 000 turnover number; Kozuch & Martin, 2012 ▸), albeit with low diastereoselectivity com­pared to that of aldehyde substrates. To determine the preferred relative stereoselectivity for the enzymatic addition into the ketone and understand how the selectivity com­pares to that with aldehyde substrates, (2*S*,3*S*)-2-amino-3-hy­droxy-3-methyl-4-phen­oxy­butanoic acid (**I**·2H_2_O) was isolated and characterized by small-mol­ecule crystallography.

**I**·2H_2_O is an example of a structure that can be equally well refined with and without taking the satellite reflections into account. The average structure model that disregards the satellite peaks meets all structural validation criteria (Spek, 2020 ▸) and refines without any indication of structural deficiencies. So does the structural model in the threefold supercell approximation. The refinement in superspace is also of good quality. The structure solution and refinement techniques of modulated structures are well established (de Wolff *et al.*, 1974 ▸, 1977 ▸; Janner & Janssen, 1977 ▸; van Smaalen *et al.*, 1995 ▸, 2004 ▸; Yamamoto, 1996 ▸; Wagner & Schönleber, 2009 ▸; Janssen, 2012 ▸; Schönleber, 2023 ▸), and are typically performed with *SUPERFLIP* (Palatinus & Chapuis, 2007 ▸), *SHELXT* (Sheldrick, 2015*a* ▸), and *JANA* (Petříček *et al.*, 2014 ▸, 2016 ▸, 2023 ▸). The number of reported modulated organic structures has been growing (Schönleber, 2011 ▸, 2023 ▸; Pinheiro & Abakumov, 2015 ▸; Brock, 2016 ▸) and in order to find other examples of structures that can be described well with all three approaches, we inter­rogated the following two databases. A manual survey of the Bilbao Incommensurate Structures Database (Aroyo *et al.*, 2006 ▸), containing 263 structures as of March 15, 2024, resulted in 23 reports of incommensurately modulated organic structures. A search of the Cambridge Structural Database (CSD; Allen, 2002 ▸; Groom & Allen, 2014 ▸; Groom *et al.*, 2016 ▸) for ‘modulated, organic only, 3D coordinates determined’ structures resulted in 11 hits. A personal correspondence with a CSD representative disclosed that whereas modulated structure entries may not be marked well and may be difficult to find, addressing this issue is on the CSD’s radar. Ultimately, it was not possible to find similar examples in the literature, but they must undoubtedly exist; numerous colleagues suggested that in the olden days of point detectors satellite reflections were likely missed, yet those structures were established and published, but the evidence seems to be anecdotal. The authors reporting modulated structures consider the average and/or supercell approximate structures when appropriate, but seldom report all three and at least one of them is usually problematic.
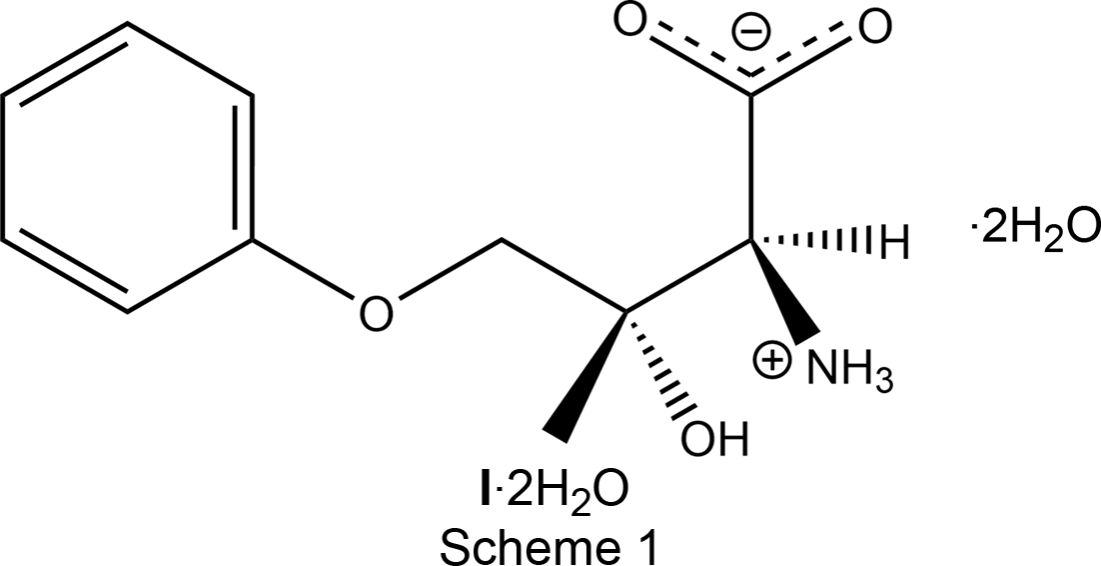


A CSD search for hydrated zwitterions relevant to **I** identified 31 com­pounds among which 10 com­pounds contained solvent water in the lattice and two had *Z*′ ≥ 3. In all 10 structures, hy­dro­gen-bonding inter­actions play an important role, but none of them is modulated. The two higher *Z*′ com­pounds are 2-ammonio-3-hy­droxy-2-(hy­droxy­meth­yl)-5-phenyl­penta­noate (*Z*′ = 3; Hernandez *et al.*, 2015 ▸) and ammonium *O*-phospho-l-threonine hydrate (*Z*′ = 4; Bryndal *et al.*, 2003 ▸). Both crystallize in Sohncke groups; in each, the symmetry-independent zwitterions exhibit different conformations and are not related by pseudosymmetry. An evaluation of the Bilbao database for incommensurate structures containing a hydrate results in several examples of incommensurate metal–organic com­plexes (Evain *et al.*, 2006 ▸; Cepeda *et al.*, 2012 ▸; Bednarchuk *et al.*, 2019 ▸; Gil-García *et al.*, 2023 ▸), but only one example of an organic com­pound where water is present as a solvent of crystallization (Rekis *et al.*, 2020 ▸, 2021 ▸). The latter article describes a superspace structure of sodium saccharinate 1.875-hydrate, in which the water mol­ecules are believed to be space filling. In contrast, the water mol­ecules in the structure presented herein play the dominant role.

The goal of the present article is to report an example of an incommensurately modulated structure that can be equally well characterized by applying the (3+1)-dimensional superspace approach, as well as average structure and supercell approximations. The occupational modulation of the positionally disordered solvent water mol­ecule in the structure of **I**·2H_2_O results from the com­petition between the two hy­dro­gen-bonding motifs that correspond to each disorder position, which are related by a small ∼0.666 (9) Å translation and ∼168 (5)° rotation about one of its O—H bonds.

## Experimental

### Single-crystal X-ray diffraction

The crystal evaluation and data collection (Table 1 ▸) were performed on a Bruker D8 Venture PHOTON III four-circle diffractometer with Cu *K*α (λ = 1.54178 Å) radiation and a detector-to-crystal distance of 5.0 cm at 100 K. The unit-cell constants for the average structure and modulated structure with one **q** vector (β = 0.357) were refined with an automated routine built into the *APEX3* program (Bruker, 2019 ▸). The data were collected using the full sphere data collection routine to survey the reciprocal space to a resolution of 0.80 Å. A total of 24 427 data were harvested by collecting 19 sets of frames with 1° scans in ω and φ, with an exposure time 1–10 s per frame. These highly redundant data sets were corrected for Lorentz and polarization effects. The absorption correction was based on fitting a function to the empirical transmission surface as sampled by multiple equivalent measurements (Krause *et al.*, 2015 ▸).

A crystal of **I**·2H_2_O was used for unit-cell determination at different temperatures in the 100–293 K range in order to detect a different unmodulated phase, but no other unit cell was discovered.

### Refinement of the average structure

A successful solution of the average structure based only on the main reflections in the space group *P*2_1_2_1_2_1_ by intrinsic phasing provided most non-H atoms from the *E* map. The remaining non-H atoms were located in an alternating series of least-squares cycles and difference Fourier maps. All non-H atoms were refined with anisotropic displacement coefficients. All H atoms attached to C atoms were included in the structure-factor calculations at idealized positions and were allowed to ride on the neighboring atoms with relative isotropic displacement coefficients.

The com­pound cocrystallizes with two solvent water mol­ecules. The O6 water mol­ecule is disordered over two positions, with a major com­ponent contribution of 0.625 (16). Both disorder com­ponents for this water mol­ecule were refined with geometrical distance restraints (Guzei, 2014 ▸). All 10 hy­dro­gen-bond donors and acceptors in the structure participate in inter­molecular hy­dro­gen-bonding inter­actions. The absolute structure was unequivocally established by anomalous dis­persion effects. The absolute configuration of both chiral atoms C2 and C3 is *S*. Visualization of the average structure and the resulting Fourier electron-density maps was done with the *OLEX2* software package (Dolomanov *et al.*, 2009 ▸).

### *NoSpherA2* refinement of the average structure

A second refinement of the average structure, using non­spherical atomic form factors, was performed using the *NoSpherA2* extension of the *olex2.refine* program (Bourhis *et al.*, 2015 ▸; Kleemiss *et al.*, 2021 ▸). The non­spherical atomic structure factors were determined by density functional theory (DFT) calculations (Neese, 2012 ▸, 2018 ▸), using the B3LYP hybrid functional and the def2-SVP basis set. All atoms were refined with anisotropic displacement coefficients. Both com­ponents for the disordered water mol­ecule were refined with geometrical (Guzei, 2014 ▸) and atomic displacement parameter restraints.

### Supercell refinement of the 1×3×1 commensurate approximate structure

Data integration and reduction were conducted in a routine fashion typical for 3D-periodic single-crystal data and only the first-order satellites were observed and taken into consideration for the supercell refinement. Due to the closeness of the **q**-vector com­ponent β = 0.357 to 1/3, indexing the reflections for a 1×3×1 supercell with a tripled *b* axis and tripled unit-cell volume of the basic cell was logical. However, generation of the supercell lowers the crystal symmetry from ortho­rhom­bic to monoclinic as the twofold screw operation along the *b* axis is lost during the conversion and now either the *a* or *c* axis could be chosen as unique. A refinement with the *c* axis unique produced better residuals and was chosen for the final supercell model in the space group *P*112_1_. *JANA2020* (Petříček *et al.*, 2023 ▸) was used to generate the mol­ecular coordinates for the supercell structure based on a 1×3×1 approximate of the superspace model.

All non-H atoms were refined with anisotropic displacement coefficients. All H atoms attached to C atoms were included in the structure-factor calculations at idealized positions and were allowed to ride on the neighboring atoms with relative isotropic displacement coefficients. All six O atoms corresponding to the disordered water mol­ecule in the average structure were refined with atomic displacement-parameter constraints. All water mol­ecules were refined with geometrical constraints (Guzei, 2014 ▸).

### Superspace refinement of the (3+1)D incommensurately modulated structure

The atomic coordinates from the average structure refinement were imported into *JANA2020* (Petříček *et al.*, 2023 ▸) in preparation for the superspace refinement of the (3+1)-dimensional structure. At the start of the refinement process, the structure was refined on *F*^2^ in the superspace group *P*2_1_2_1_2_1_(0β0)000 (β = 0.357) using only the main reflections to establish a baseline ‘average’ structure refinement following importation into *JANA2020*. Once the modulation wave parameters were introduced to the model, both satellite and main reflections were taken into consideration and the instability factor was calculated from the reflection statistics. The average structure could also be solved independently with either *SUPERFLIP* (Palatinus & Chapuis, 2007 ▸) or *SHELXT* (Sheldrick, 2015*a* ▸) within *JANA2020* in a straightforward manner.

The structure was refined as an inversion twin, with a minor com­ponent contribution of 3(8)%. All non-H atoms were refined with anisotropic displacement coefficients, while all carbon-bound H atoms were placed in idealized positions and allowed to ride on neighboring atoms with relative isotropic displacement coefficients. The remaining H atoms (those bound to N or O atoms) were refined with geometric and atomic displacement-parameter restraints in a manner con­sis­tent with the *SHELXL* refinement of the average structure. Unlike the average structure refinement, however, both or­dered and disordered water mol­ecule geometries were restrained based on a DFT-optimized geometry (Guzei, 2014 ▸).

The displacive modulation for all atoms in the zwitterion and the ordered O5 water mol­ecule was described with one harmonic modulation wave and the anisotropic displacement parameter (ADP) modulation for the non-H atoms in these mol­ecules was also described with one harmonic modulation wave. The refinement of the occupational and positional modulations of the disordered water mol­ecule (the major disorder com­ponent is labeled O6 and the minor O7) was problematic and several models were explored (see next paragraph). The best superspace refinement results from a model where the O6 and O7 water mol­ecules are treated as rigid bodies (centered on the O atoms), the occupational modulation is described with a second-order harmonic function (where the occupancy modulation of the major and minor disorder com­ponents is constrained to be com­plementary), and the positional modulation is described with a first-order harmonic function. All attempts to model the ADP modulation of atoms O6 and O7 resulted in non­positive-definite *U^ij^* tensors; thus, these atoms were refined without ADP modulation. Visualization of the modulated structure and the associated Fourier electron-density map was performed using the *JanaDraw* and *RunContour* extensions in *JANA2020* (Petříček *et al.*, 2023 ▸), respectively.

*Additional refinement details:* The rigid-body approach for the refinement of the disordered water mol­ecule was deemed necessary because the displacive modulations of the riding H atoms could not be reliably refined independently. Refinement of the occupancy modulation using only first-order harmonics resulted in unrealistic values (up to 112% for O6 and as low as −12% for O7; see Fig. S2 in the supporting information); thus, we turned to a refinement where the first and second-order satellites were treated as overlapped reflections, allowing for the implementation of a second-order harmonic function for the occupancy modulation of the disordered water mol­ecules. However, attempts to refine the positional modulation for either the ordered or disordered atoms with second-order harmonics led to instabilities in the refinement, likely due to the com­paratively weak contribution of the displacive modulation com­ponent to the overall behavior of the structure; thus, the use of a first-order harmonic was considered sufficient.

Refinement of the occupational modulation of the disordered water mol­ecule with a crenel function was also ex­plored. The magnitude of delta corresponding to the average occupancy of the O6 atom refined to a value of 0.6659, which is slightly larger than those resulting from the refinement of the average structure in *SHELXL* (0.625) and *JANA* (0.631). Indeed, visualization of this discontinuous occupancy function for atom O6 over an inter­val of *t* and overlayed with the electron-density map clearly reveals this value to be an overestimation of the occupancy of the O6 mol­ecule (Fig. S1). A refinement where the delta value for the water mol­ecule containing atom O6 was constrained to be equal to the occupancy value of the O6 atom in the refinement of the average structure in *JANA* was considered, but it was decided that such an approach was not satisfying or well justified.

### Density functional theory (DFT) calculations

The geometry of **I** was optimized with *GAUSSIAN16* (Frisch *et al.*, 2016 ▸) at the B3LYP/6-311+G(d,p) level of theory with the polarizable continuum model for implicit aqueous solvation.

### Synthetic procedure

The synthetic procedure is reported by Bruffy *et al.* (2023 ▸).

## Structure solution and refinement

**Figure 1:**
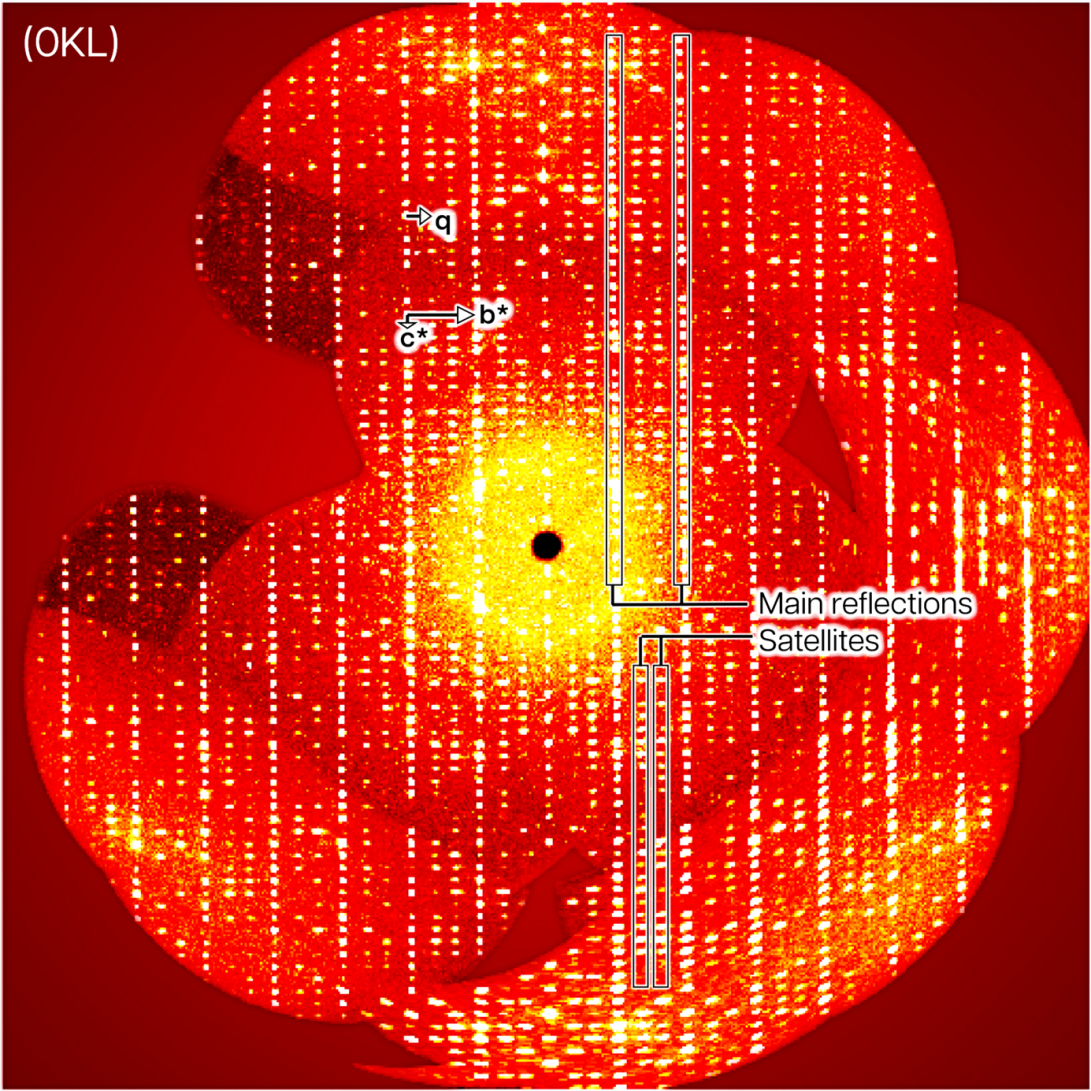
Annotated reconstruction of the 0*kl* reciprocal space layer for **I**·2H_2_O(mod).

The diffraction pattern (Fig. 1 ▸) clearly shows the presence of strong main and weaker satellite reflections (Table 2 ▸). The reflections were indexed for three different refinement models as follows: (i) with three *hkl* Miller indices considering the main reflections only for the average structure solution and refinement; (ii) with three Miller indices only in the superstructure (*V* = 3787 Å^3^) that is based on both main and satellite reflections; (iii) with three *hkl* Miller indices and one **q**-vector in the superspace model based on the main and first-order satellite reflections.

### Average structure I·2H_2_O(av)

**Figure 2:**
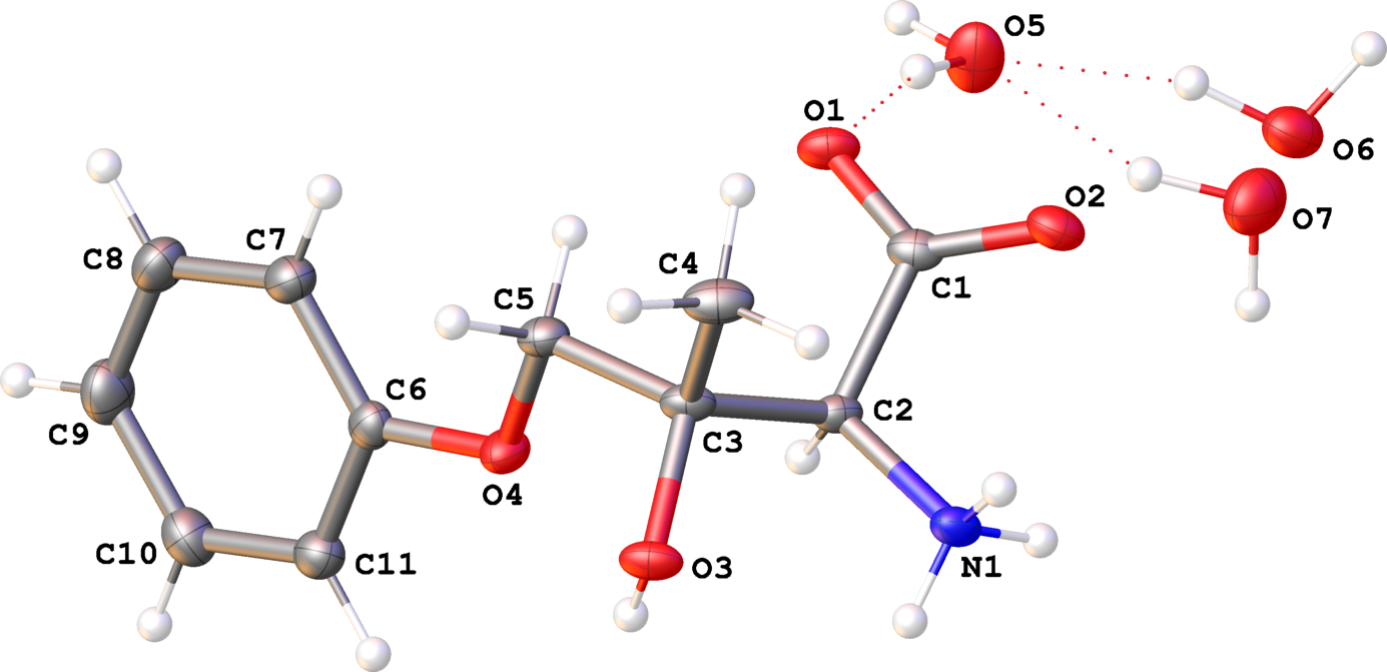
Mol­ecular drawing of the asymmetric unit of the average structure **I**·2H_2_O(av), shown with 50% probability displacement ellipsoids. The O6 water mol­ecule is present 62.5 (16)% of the time and the O7 water mol­ecule 37.5 (16)% of the time. The configuration of both chiral atoms C2 and C3 is *S*.

The zwitterion **I** crystallizes with a proton transferred from the carb­oxyl group to amine atom N1 and two water solvent mol­ecules in the asymmetric unit (Fig. 2 ▸). The absolute structure [Hooft *y* = 0.02 (3)] and absolute configuration (*S* for both C2 and C3) were unequivocally established by resonant scattering effects. All bond distances and inter­atomic bond angles fall in the usual ranges, as confirmed by a *Mogul* (Bruno *et al.*, 2004 ▸) geometry check, with a possible exception of the carboxyl­ate atoms O1, O2, and C1 being coplanar with atoms N1 and C2 within 0.027 Å. The arene ring and atom O4 are coplanar within 0.015 Å, but atoms C3 and C5 are displaced by 0.26 (5) Å from this plane. One water mol­ecule (O5) is fully occupied and ordered, whereas the other is disordered over positions O6 and O7 in a 0.625 (16):0.375 (16) ratio.

**Figure 3:**
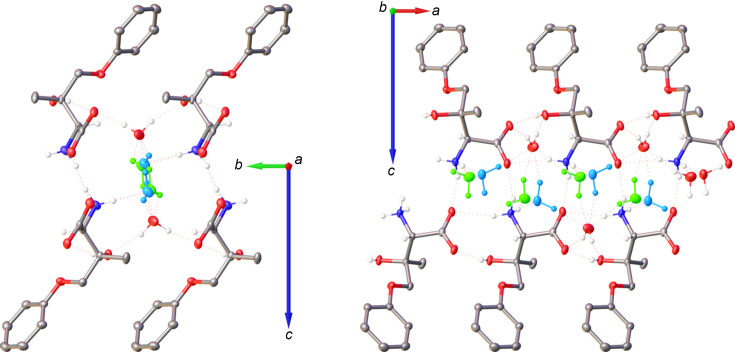
Mol­ecular drawings highlighting the hy­dro­gen-bonding network in the average structure **I**·2H_2_O(av), shown along the *a* axis (left) and the *b* axis (right), with 50% probability displacement ellipsoids. The partially occupied O6 and O7 water mol­ecules are shown in blue and green. All H atoms that do not participate in hy­dro­gen-bonding inter­actions and are not bound to chiral atoms have been omitted.

There are five hy­dro­gen-bond donor atoms with 10 H atoms among them; all ten H atoms participate in inter­molecular hy­dro­gen-bonding inter­actions. These bonds are of the types O—H⋯O and N—H⋯O, and range from weak to strong and charge-assisted, with *D*—H⋯*A* distances ranging between 2.7071 (17) and 3.0237 (18) Å, with the *D*—H⋯*A* angles falling in the 139 (5)–173 (2)° range. The important part of the hy­dro­gen-bonding network involves the ammonium N1 atom and both water mol­ecules. The ammonium group forms a bond to the disordered water mol­ecule (either N1—H1*C*⋯O6 or N1—H1*C*⋯O7), which in turn makes a hy­dro­gen-bonding inter­action with one of its H atoms with the ordered O5 water mol­ecule (O6—H6*B*⋯O5 and O7—H7B⋯O5). However, the other H atoms on each partially occupied water mol­ecule point in the opposite directions and form bonds to two different ordered water mol­ecules: the higher populated site O6 forms the stronger bond O6—H6*A*⋯O5(*x* + 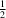, −*y* + 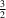, −*z* + 1), with *D*⋯*A* = 2.980 (7) Å and *D*—H⋯*A* = 154 (3)°, whereas the less populated site is characterized with a shorter O7—H7*A*⋯O5(*x* − 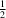, −*y* + 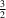, −*z* + 1) distance of *D*⋯*A* = 2.849 (12) Å and a suboptimal *D*—H⋯*A* angle of 139 (5)° (Fig. 3 ▸). The hy­dro­gen bonds form ∼7.2 Å-thick two-dimensional networks parallel to the *ab* plane and are separated by hydro­phobic layers along the *c* direction.

**Figure 4:**
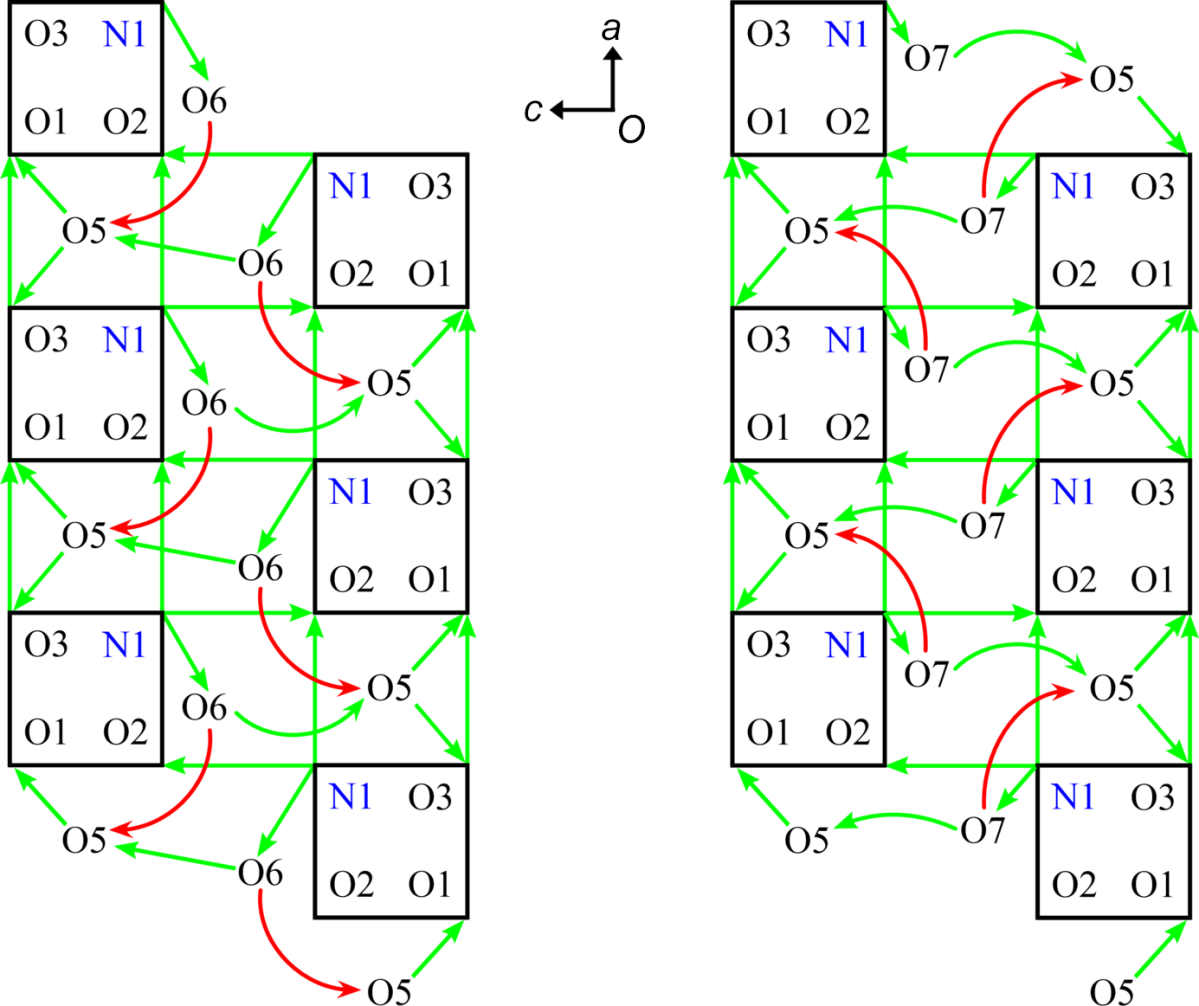
A schematic representation of hy­dro­gen-bonding inter­actions in **I**·2H_2_O(av). Each mol­ecule of **I** is represented with a square with each corner marked with an atom label corresponding to a hy­dro­gen bond donor (*D*) or acceptor (*A*). The arrows extend from *D*—H donors to acceptors: *D*→*A*.

The hy­dro­gen-bonding inter­actions are shown in Fig. 4 ▸, similar to the approach of Savic *et al.* (2021 ▸). The mol­ecules of **I** are linked into hy­dro­gen-bonded columns along the *a* direction by an 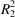
(8) motif N1→O2⋯O1←O3, which is seen in each column of mol­ecules in Fig. 3 ▸. Each column is connected to a column related by 2_1_ with hy­dro­gen-bonding 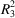
(9) motifs N1→O2←N1⋯O2←N1, observed between the mol­ecular columns in Fig. 3 ▸; these columnar dimers propagate in the *a* direction. The columnar dimers are connected in the *b* direction by solvent water mol­ecules into two-dimensional sheets perpendicular to *c* as follows. The water mol­ecules O5 connect two columns of **I** related by a 1,0,0 translation into dimeric columns along *b* with a 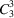
(6) motif O1←O5→O3→O1. In Fig. 4 ▸, these inter­actions appear as O1←O5→O3→O1 triangles, but they are spirals because the O5 atoms connect mol­ecules in different layers perpendicular to the plane of the paper (*b* direction) rather than in the plane of the paper. The water mol­ecules O6 form three hy­dro­gen bonds and so do the mol­ecules of O7 (Fig. 4 ▸). Two of their inter­actions are to the same atoms, O6→O5 and O6←N1, and O7→O5 and O7←N1, correspondingly. Their third bonds differ, O6→O5′ *versus* O7→O5′′, and are shown in red to emphasize the difference. At this point, further graph-set notation descriptions of the hy­dro­gen-bonding network in the hydro­phobic layers parallel to the *ab* plane becomes impractical.

### Average structure refined with *NoSpherA2* I·2H_2_O(NS2)

**Figure 5:**
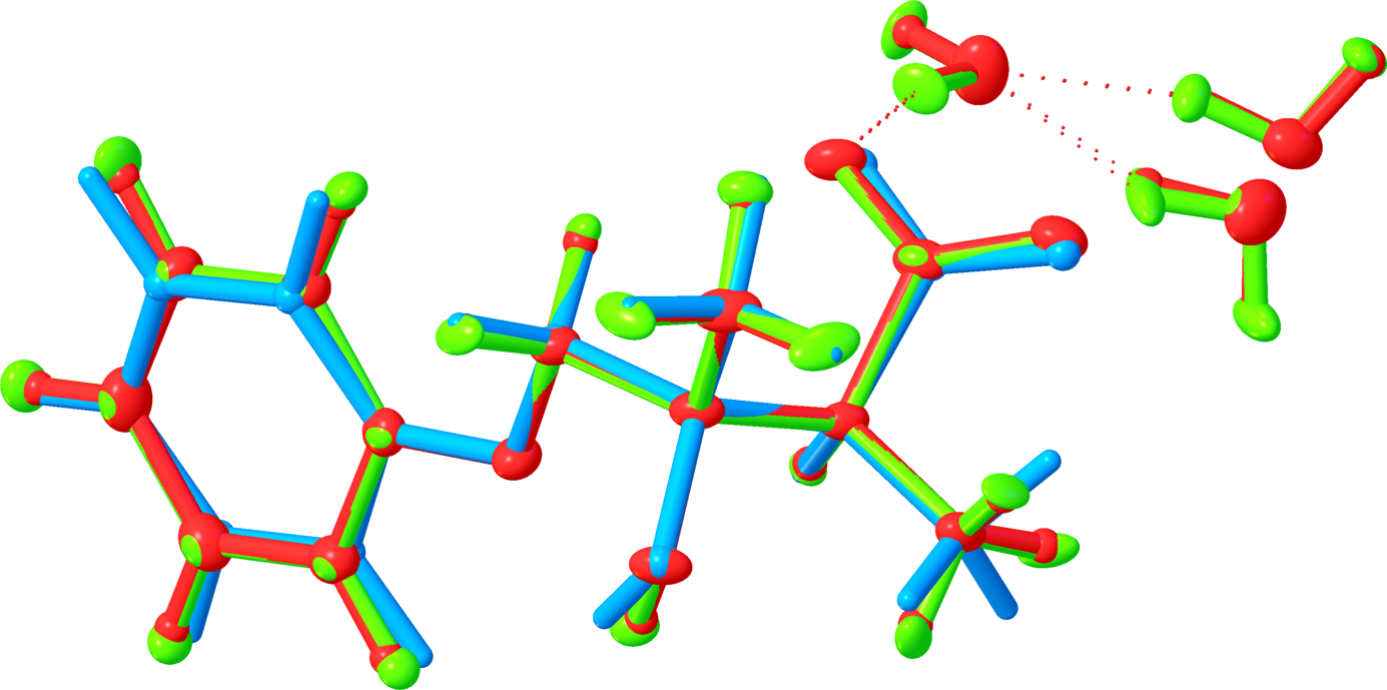
Overlay of **I**·2H_2_O(av), **I**·2H_2_O(NS2), and **I**-DFT shown in red, green, and blue, respectively.

The structural refinement of the average structure with a non­spherical atom model, as implemented in the *NoSpherA2* extension of the *olex2.refine* program (Kleemiss *et al.*, 2021 ▸; Bourhis *et al.*, 2015 ▸), produces lower *R* factors and a more precise model with standard deviations on the inter­atomic bond distances two to three times smaller than those in **I**·2H_2_O(av). These improvements come at the cost of a lower data-to-parameter ratio [7.08 for **I**·2H_2_O(NS2) *versus* 12.6 for **I**·2H_2_O(av)], as both non-H and H atoms are refined anisotropically. In **I**·2H_2_O(NS2), the C—H and N—H distances are expectedly longer than the corresponding distances in **I**·2H_2_O(av), but other distances show minor variations and the non-H-atom geometries of **I**·2H_2_O(av) and **I**·2H_2_O(NS2) can be superimposed with a root mean square deviation (RMSD) of 0.004 Å (Fig. 5 ▸).

### Density functional theory (DFT) calculations (I-DFT)

The geometry of **I**-DFT closely matches the experimentally observed conformation; the non-H atoms of the mol­ecule could be superimposed onto **I**·2H_2_O(av), with RMSD = 0.123 Å (Fig. 5 ▸). The main differences are in the relative orientations of the arene ring and carb­oxyl group: the dihedral angle between the arene planes in the superimposed **I**·2H_2_O(av) and **I**-DFT is 8.8°, whereas the dihedral angle between the O1/O2/C1 planes measures 7.74°. In contrast to **I**·2H_2_O(av), atoms C3 and C5 in **I**-DFT are nearly coplanar with the phenolate fragment.

A single-point energy calculation for the experimental geometry of **I**·2H_2_O(NS2) reveals that its conformation is 9.8 kcal mol^−1^ higher than that of **I**-DFT. This may be in part due to suboptimal element–hy­dro­gen distances, in part due to the omission of the water mol­ecules, and in part due to lattice effects that stabilize the observed conformation of **I** that facilitates the formation of strong charge-assisted hy­dro­gen bonds.

### Supercell and superstructure model I·2H_2_O(supercell)

Another approximation for the solid-state description of the incommensurately modulated **I**·2H_2_O is the refinement of its superstructure in the supercell. Van Smaalen stated that a supercell refinement may be com­parable to that of the superspace model when satellites of the first order only are taken into consideration (van Smaalen *et al.*, 1995 ▸). Indeed, this supercell approximation confirmed a strong occupational modulation of the disordered water mol­ecules and a small positional modulation of both the zwitterion **I** and the ordered water mol­ecule.

**Figure 6:**
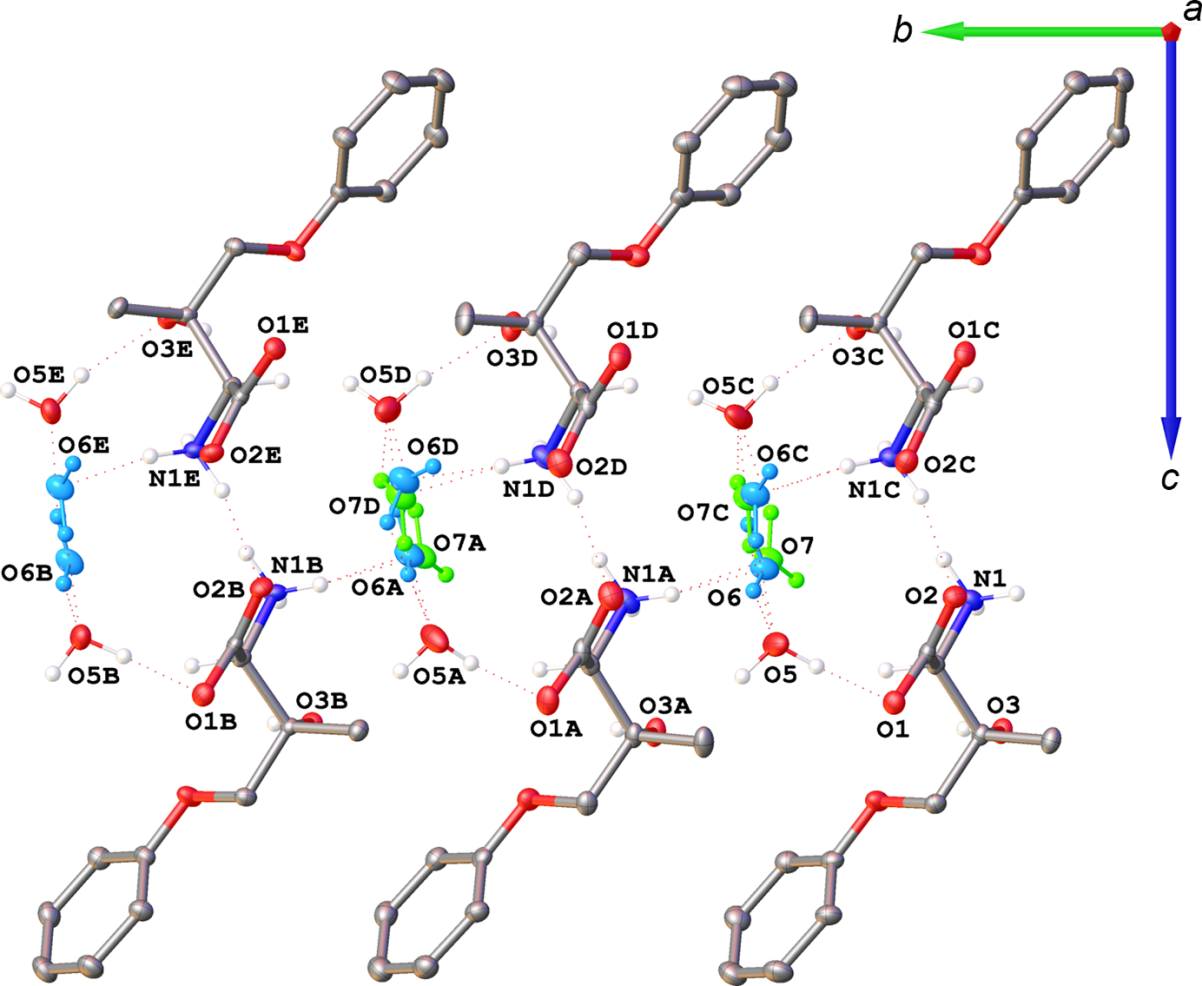
Mol­ecular drawing of **I**·2H_2_O(supercell), shown with 50% probability displacement ellipsoids. The six symmetry-independent sites in the supercell corresponding to the disordered O6 and O7 water mol­ecules are shown in blue and green. All H atoms that do not participate in hy­dro­gen-bonding inter­actions and are not bound to chiral atoms have been omitted.

In the supercell approximation, the threefold lengthening of the *b* axis and lowering of the point-group symmetry from 222 to 2 resulted in *Z*′ = 6 with six symmetry-independent mol­ecules of **I** and 12 mol­ecules of solvent water (Fig. 6 ▸). In the average structure, one water mol­ecule is ordered and one disordered; therefore, in the supercell, the expectation was to observe six sites with ordered water mol­ecules and six with disordered ones. This was not the case. The former six water mol­ecules are ordered, but among the six sites for the latter six mol­ecules, two contain ordered water mol­ecules and four are occupied by disordered water mol­ecules with two disorder ratios (Table 3 ▸). These differences in the occupational parameters must be the reason why the symmetry along the *b* axis is lost in the supercell.

**Figure 7:**
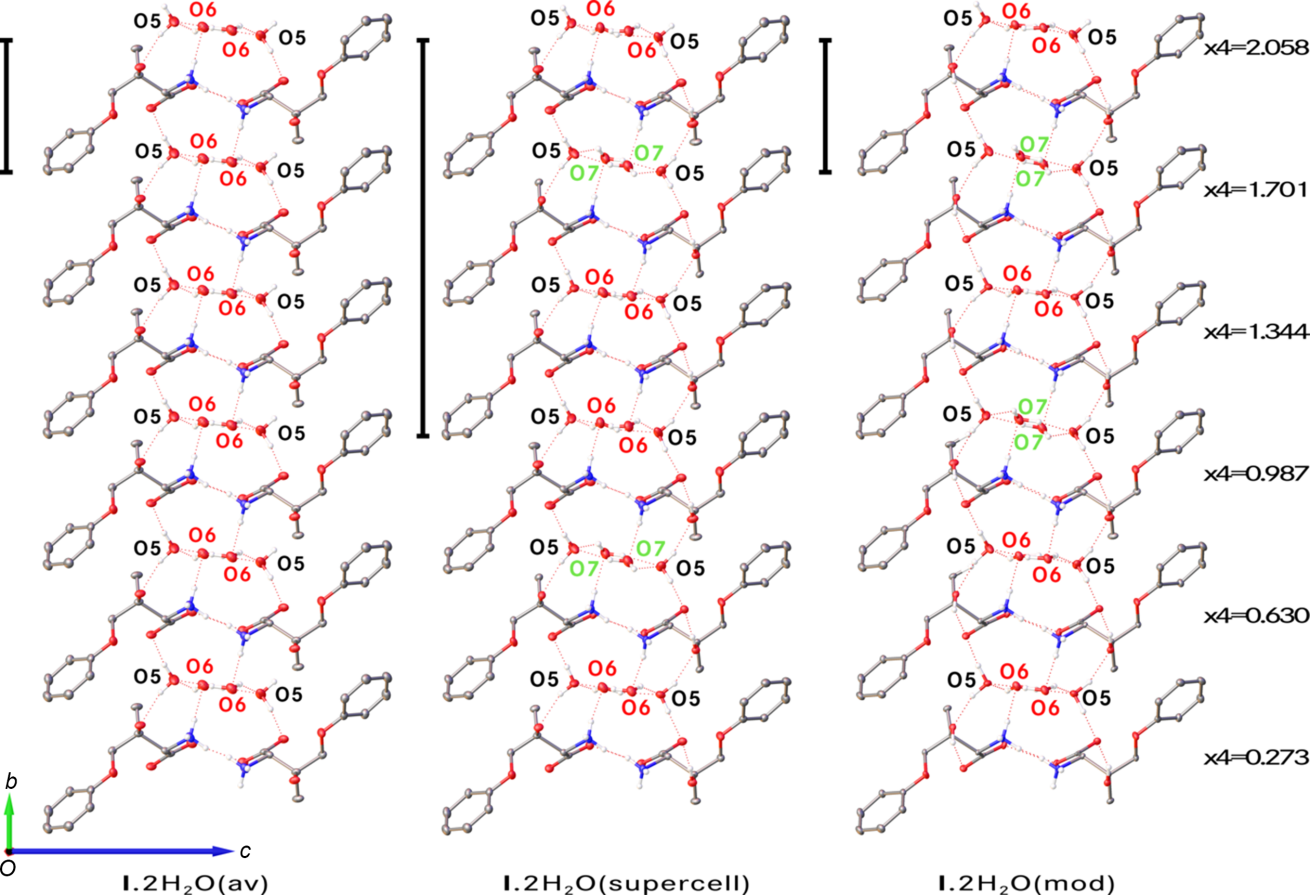
Mol­ecular packing diagrams for **I**·2H_2_O(av), **I**·2H_2_O(supercell), and **I**·2H_2_O(mod). Water mol­ecules are shown if their occupancies exceed 50%. Only water mol­ecules are labeled and all H atoms on C atoms have been omitted. The vertical bars represent the lengths of the *b* axes. For the **I**·2H_2_O(mod), the *t* = 0 corresponds to the O6 atom coordinate *y* = 0.76343.

These occupational percentages are explained with the help of Table 3 ▸ that lists them for the six sites of the expected disorder. The occupancies follow a sawtooth distribution both for O6 and for O7, with an average of 0.635 for the main disorder com­ponent. Whereas this number is in excellent agreement with the value of 0.625 (16) observed for **I**·2H_2_O(av) and 0.624 (3) for **I**·2H_2_O(mod), the individual occupancies at the six sites must be different in the crystal because the supercell refinement is an approximation due to the modulation wavelength being 2.8*b* (17.70 Å) rather than 3*b* (18.97 Å) exactly. It is instructive to com­pare the relative distribution of the occupancy factors of the disordered water mol­ecules among **I**·2H_2_O(av), **I**·2H_2_O(supercell), and **I**·2H_2_O(mod). Fig. 7 ▸ shows mol­ecular arrangements along the *b* direction for the three models, but the disordered O6 and O7 mol­ecules are shown only when their occupancy exceeds 50% to demonstrate the differences in the modeled occupancy factors.

**Figure 8:**
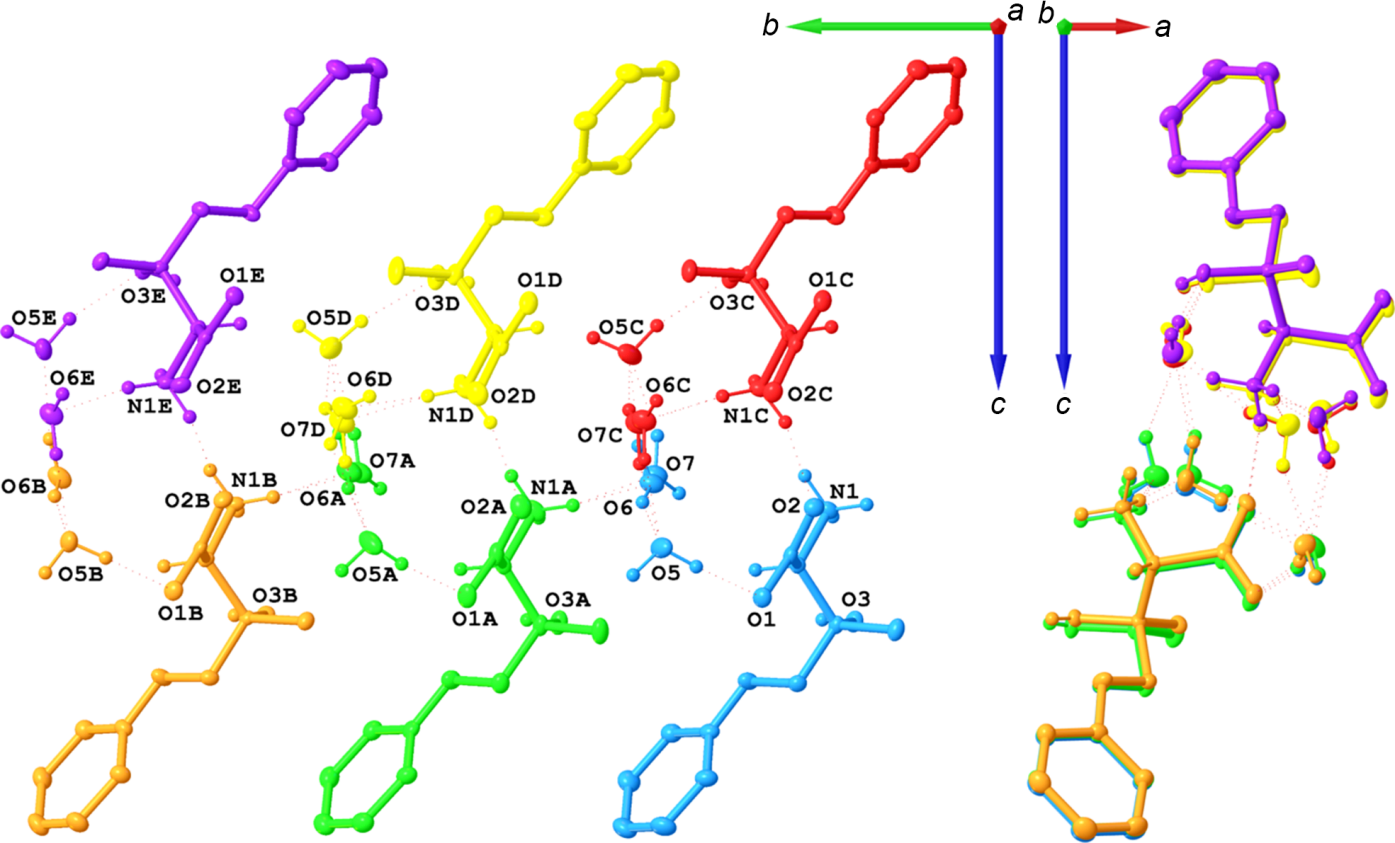
Mol­ecular drawing of **I**·2H_2_O(supercell), shown with 50% probability displacement ellipsoids along the *a* and *b* directions. The mol­ecule without a label suffix is shown in blue, while the mol­ecules with label suffixes *A*, *B*, *C*, *D*, and *E* are shown in green, orange, red, yellow, and purple, respectively. All H atoms that do not participate in hy­dro­gen-bonding inter­actions and are not bound to chiral atoms have been omitted.

**Figure 9:**
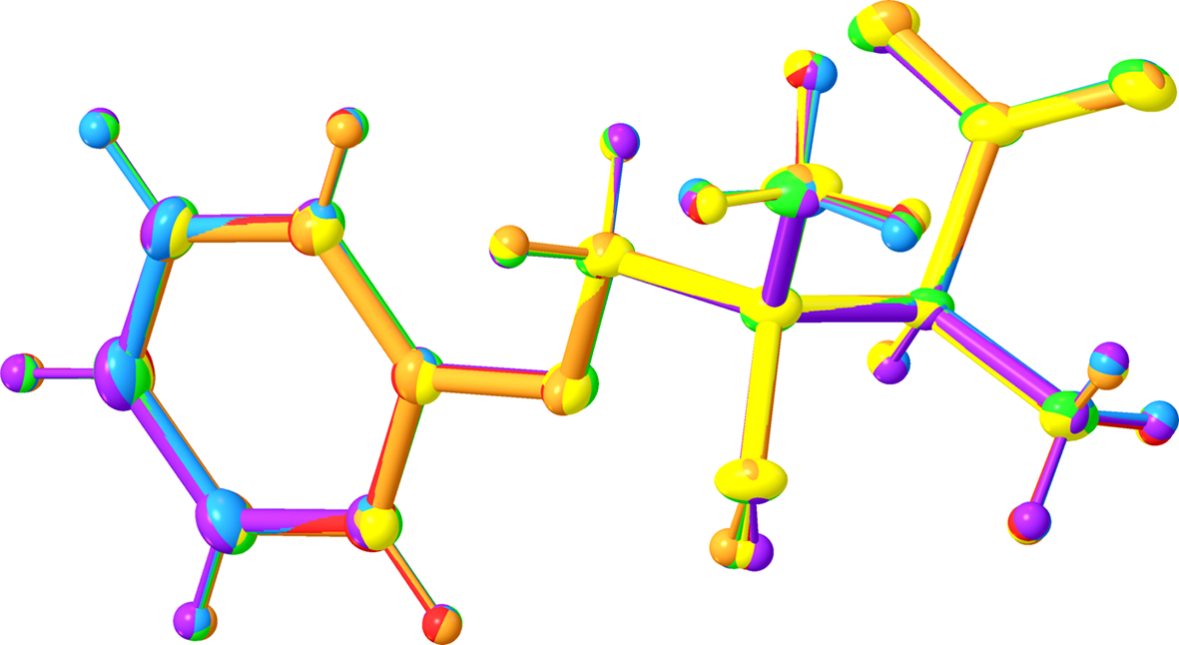
Overlay of the six symmetry-independent mol­ecules of **I** in the structure of **I**·2H_2_O(supercell), shown with 50% probability displacement ellipsoids. The mol­ecules are matched by a least-squares fitting routine. The mol­ecule without a label suffix is shown in blue, while the mol­ecules with label suffixes *A*, *B*, *C*, *D*, and *E* are shown in green, orange, red, yellow, and purple, respectively.

Fig. 8 ▸ highlights how viewing the six symmetry-independent mol­ecules along the *b* direction gives an impression of the amplitude of the displacive modulation described with the superspace approach in Section 3.5. In the structure of **I**·2H_2_O(supercell), the six mol­ecules of **I** have very similar geometries: mol­ecules with label suffixes *A*, *B*, *C*, *D*, and *E* can each be superimposed (with the H atoms included) onto the mol­ecule without a label suffix with RMSDs of 0.016, 0.053, 0.050, 0.057, and 0.037 Å, respectively (Fig. 9 ▸).

### Superspace model I·2H_2_O(mod)

**Figure 10:**
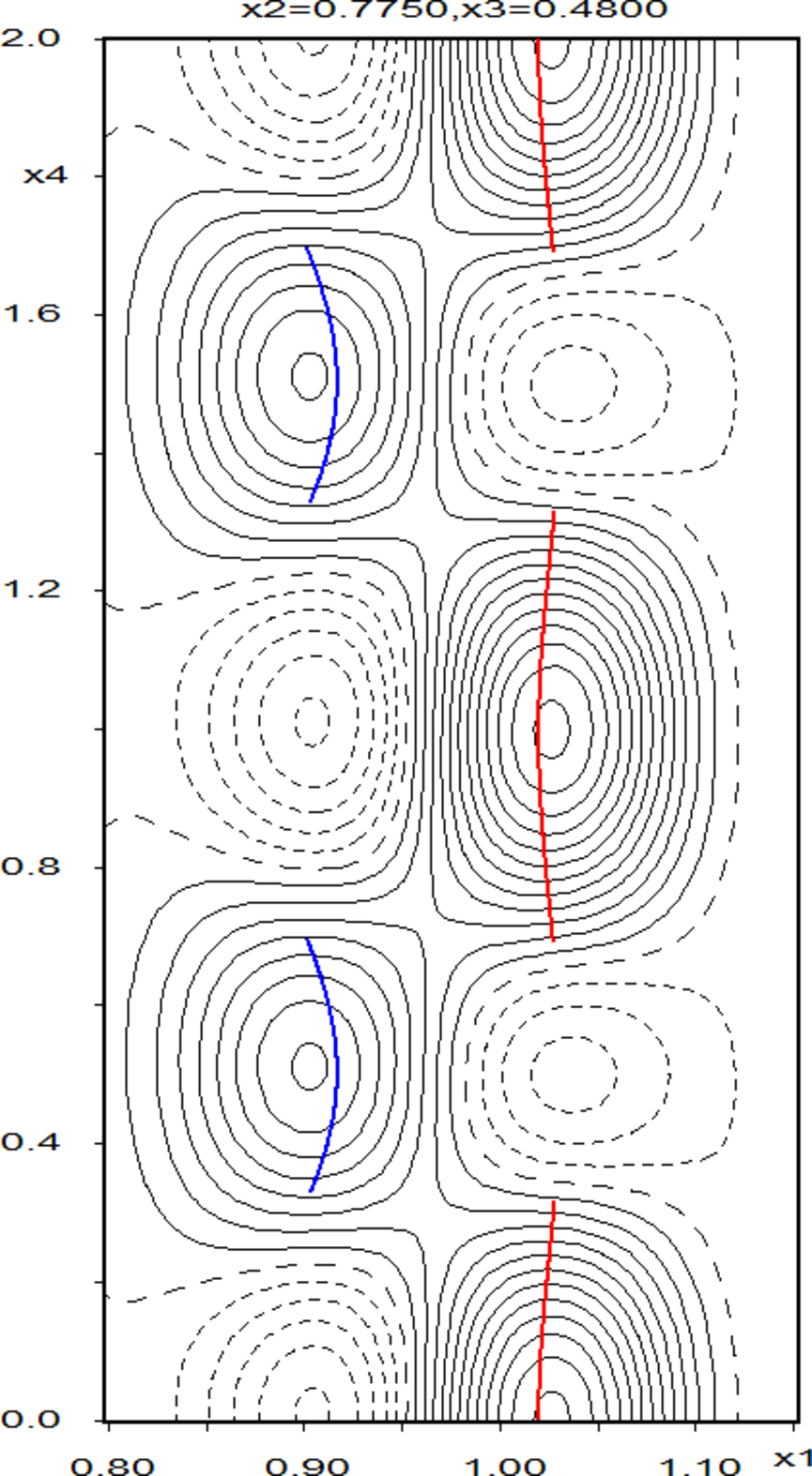
Electron-density plot showing the (3+1)D modulation of the O6 and O7 atoms along *x*1 as a function of *x*4, with the electron density summed over a thickness of 1 Å in the remaining directions. Solid and dashed black lines represent areas of positive and negative electron density. The curves of the red and blue lines represent the paths of motion of the O6 and O7 atoms. This occupational modulation is manifested as positional disorder where the water mol­ecule is split over the O6 and O7 sites, which have an average occupancy ratio of 62.4 (3):37.6 (3) in **I**·2H_2_O(mod).

The (3+1)D superspace approach is the most accurate way to describe the structure of **I**·2H_2_O. Our final model, using two harmonic waves for the occupancy modulation of atoms O6 and O7 (Fig. 10 ▸), results in an average partial occupancy equal to 0.624 (3) for atom O6, which is nearly identical to the partial occupancy of O6 [0.625 (16)] obtained from the average structure refinement in *SHELXL*.

For the most part, the zwitterion moves as a rigid unit with a small displacement amplitude in the *a* and *b* directions, while in the *c* direction, all atoms move in a highly concerted fashion with a slightly larger degree of displacement, where the largest amplitude corresponds to the terminal C4 methyl group (∼0.15 Å). The exception occurs in the *b* direction, where the planar phenolate region exhibits a swaying motion corresponding to a 4.39 (11)° rotation of the arene ring about the O4—C6 bond. The amplitude of this motion (0.07 < dy < 0.12) is noticeably larger than that for the remaining regions of **I** in the *b* direction (where atom C5 has the largest amplitude of ∼0.05 Å). Atom N1 also exhibits a pronounced displacement amplitude in the *b* direction (∼0.08 Å), which likely results from the participation of the atom in hy­dro­gen-bonding inter­actions. Plots showing the atomic displacement functions *versus t* for all non-H atoms are provided in Fig. S4 of the supporting information.

**Figure 11:**
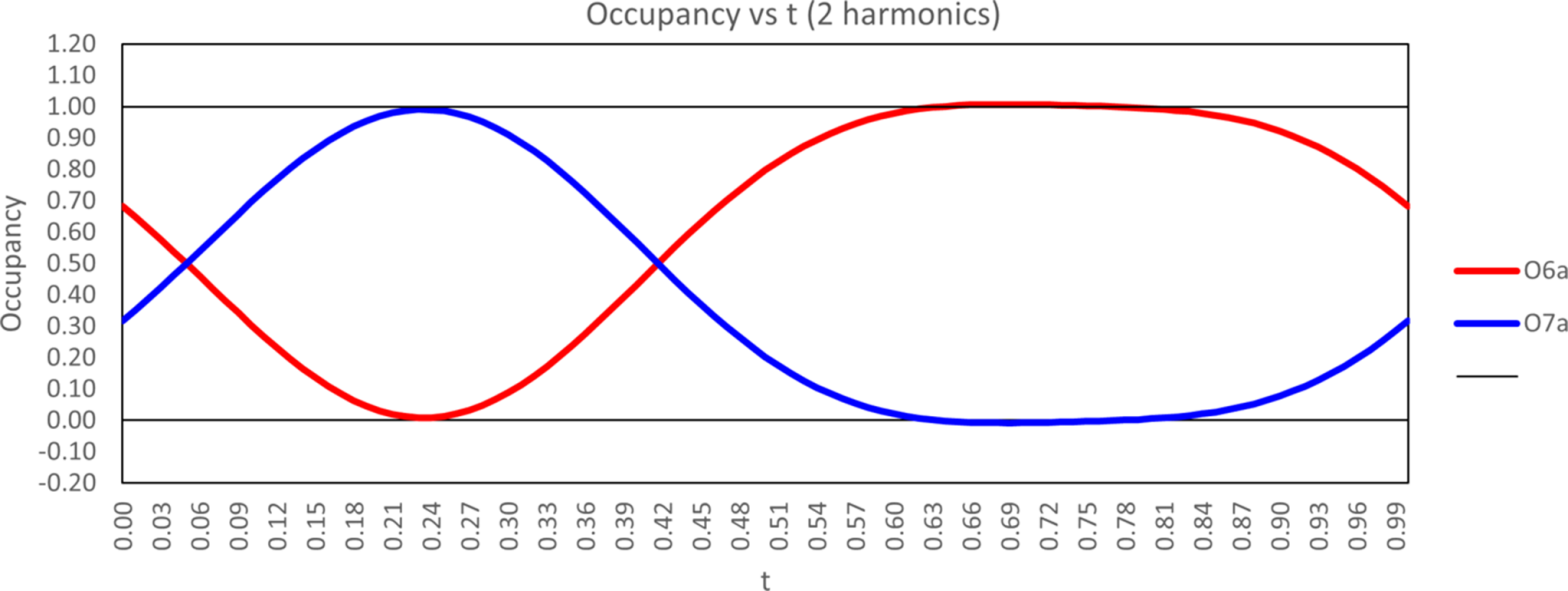
Plot of the two harmonic modulation functions used to model the occupancy modulation of atoms O6 and O7 *versus t*.

Overall, the amplitudes of the displacive modulation of **I**·2H_2_O(mod) is small in all three directions (≤ 0.15 Å) and likely just a response to the occupational modulation of the disordered water mol­ecule, which manifests itself as two orientations, with the O6—H6*B* and O7—H7*B* bonds pointing along the same direction in *c*, and the O6—H6*A* and O7—H7*A* bonds pointing in opposite directions along *a*. Neither orientation allows for perfectly optimized hy­dro­gen-bonding inter­actions. In terms of the *D*⋯*A* distance, the O7 orientation appears to be favored over the O6 orientation [average *D*⋯*A* = 2.757 (19) Å *versus* 2.983 (4) Å for O7⋯O5^v^ and O6⋯O5^iv^; symmetry codes: (iv) *x* + 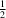, −*y* + 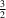, −*z* + 1; (v) *x* − 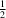, −*y* + 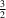, −*z* + 1]. Meanwhile, in terms of the *D*—H⋯*A* angle, the O6 orientation appears to be favored [average *D*—H⋯*A* = 159 (4)° *versus* 140 (7)° for O6—H6*A*⋯O5^iv^ and O7—H7*A*⋯O5^v^]. Thus, the occupational modulation likely arises from the com­petition between these hy­dro­gen-bonding inter­actions. In fact, a plot of the occupational modulation *versus t* nicely aligns with how these hy­dro­gen-bonding inter­actions fluctuate (Fig. 11 ▸). Specifically, the occupancy of O6 is lowest over the inter­val of 0.06 < *t* < 0.4, which is the same range during which the O6⋯O5^iv^ distance is the least optimized, while the occupancy of O7 is lowest over the inter­val of 0.6 < *t* < 0.9, which is the same range during which the O7—H7*B*⋯O5 and O7—H7*A*⋯O5^v^ angles are least optimized.

The N1^iv^—H1*B*^iv^⋯O6 and N1^iv^—H1*B*^iv^⋯O7 hy­dro­gen-bond inter­actions follow a similar pattern, where the *D*⋯*A* distances are always slightly shorter for N1^iv^⋯O7 than for N1^iv^⋯O6 throughout the full range of *t* values, while the *D*—H⋯*A* angles for N1^iv^—H1*B*^iv^⋯O6 are better optimized com­pared to N1^iv^—H1*B*^iv^⋯O7 [170.8 (14)–173.8 (14) *versus* 159.7 (15)–169.6 (15)°] over the full range of *t* values. Specifically, the values for the N1^iv^⋯O6 and H1*B*^iv^⋯O6 distances, as well as the N1^iv^—H1*B*^iv^⋯O6 angle, are the least optimized within the region of 0.06 < *t* < 0.4 (where the occupancy of atom O6 approaches zero and the occupancy of atom O7 approaches 1), while the values for the H1*B*^iv^⋯O7 distance and the N1^iv^—H1*B*^iv^⋯O7 angle are now the most optimized within the same range of *t* values. Plots showing the described hy­dro­gen-bonding inter­actions *versus t* are provided in Figs. S5 and S6 of the supporting information.

The observed modulation emphasizes the role of hydrogen-bonding in the stabilization of the structure and is attributed to the occupational modulation of the disordered water mol­ecule O6/O7. The inter­molecular inter­actions formed by these partially occupied water mol­ecules do not conform to the 3D space-group symmetry operations. Whereas a dynamical disorder between these two positions is possible due to the sufficient room in this void to allow, for example, mol­ecule O6 to rotate about one of its O—H bonds and slide into the position of O7, it would be unlikely because there are no hy­dro­gen-bond acceptors for the transition geometries of this mol­ecule. Competition between the hy­dro­gen bonding and preferred conformation of **I** does not seem to be a major reason because the geometry of **I** does not change with the modulation, but its orientation changes slightly. Interplay between the strong hy­dro­gen bonding and optimal mol­ecular packing may give rise to the loss of 3D symmetry, but again large displacive modulations in **I**·2H_2_O(mod) are not ob­served. The mol­ecules pack with alternating hy­dro­gen-bonded hydro­philic and hydro­phobic layers, with no π–π inter­actions in the lattice.

## Conclusions

The average, commensurate supercell, and incommensurate superspace refinements provide adequate and com­parable descriptions of **I**·2H_2_O, but with varying levels of detail. In all three models, the disorder ratio for two positions of the disordered water mol­ecule refines to essentially the same value, *i.e.* 0.63:0.37. The modulation in the superspace structure is characterized as moderate due to the strength of the first-order satellite reflections. The superspace refinement was problematic due to com­putational instabilities, occupational modulation, and possible satellite reflection overlap. The average structure provides a benchmark for the disorder refinement, and its high quality make it easy to overlook the modulation. An average structure refinement with non­spherical atom form factors did not uncover any structural problems. The supercell approximation reveals a more com­plicated nature of the positional disorder of the water mol­ecule, and the superspace refinement clarifies the nature of the com­petition between the different hy­dro­gen-bonding inter­actions of O6 and O7. There is a strong correlation between the behavior of the occupancy modulation of the positionally disordered O6/O7 water mol­ecule and the optimization of the hy­dro­gen-bonding motifs associated with each position.

## Supplementary Material


9GYXSFst2pE

Crystal structure: contains datablock(s) buller05a_AVG, buller05a_NS2, buller05a_supercell, buller05a_MOD, global. DOI: 10.1107/S2053229624007009/op3030sup1.cif

Structure factors: contains datablock(s) buller05a_NS2. DOI: 10.1107/S2053229624007009/op3030buller05a_NS2sup2.hkl

Structure factors: contains datablock(s) buller05a_AVG. DOI: 10.1107/S2053229624007009/op3030buller05a_AVGsup3.hkl

Structure factors: contains datablock(s) buller05a_supercell. DOI: 10.1107/S2053229624007009/op3030buller05a_supercellsup4.hkl

Supporting information file. DOI: 10.1107/S2053229624007009/op3030buller05a_AVGsup5.cml

Supporting information file. DOI: 10.1107/S2053229624007009/op3030sup6.pdf

Individual CIFs. DOI: 10.1107/S2053229624007009/op3030sup7.zip

CCDC references: 2371542, 2371541, 2371540, 2371539

## Figures and Tables

**Table 1:** Crystallographic experimental details

	**I**·2H_2_O(av)	**I**·2H_2_O(NS2)	**I**·2H_2_O(supercell)	**I**·2H_2_O(mod)
Crystal data				
*M*_r_	261.27	261.28	261.27	261.3
Crystal system, space group	Ortho­rhom­bic, *P*2_1_2_1_2_1_		Monoclinic, *P*112_1_	Ortho­rhom­bic, *P*2_1_2_1_2_1_(0β0)000 β = 0.357
*a*, *b*, *c* (Å)	5.6620 (5), 6.3235 (5), 35.277 (3)		5.6737 (11), 19.021 (4), 35.298 (7)	5.662 (2), 6.324 (2), 35.276 (7)
γ (°)			90.030 (7)	
*V* (Å^3^)	1263.05 (18)		3809.4 (12)	1263.1 (6)
*Z*	4		12	4
Radiation type	Cu *K*α			
Temperature (K)	100			
μ (mm^−1^)	0.95			
Crystal size (mm)	0.18 × 0.16 × 0.11			
				
Data collection				
*T*_min_, *T*_max_	0.674, 0.754		0.678, 0.754	0.678, 0.754
No. of measured, independent, and observed reflections	24427, 2563, 2552 [*I* > 2σ(*I*)]		84909, 15929, 14589 [*I* > 2σ(*I*)]	28094, 7937, 7346 [*I* > 3σ(*I*)]
*R*_int_	0.026		0.049	0.028
(sin θ/λ)_max_ (Å^−1^)	0.633			
				
Refinement				
*R*[*F*^2^ > 2σ(*F*^2^)], *wR*(*F*^2^), *S*	0.025, 0.068, 1.08	0.012, 0.030, 1.12	0.053, 0.158, 1.07	0.0364, 0.0956, 2.203
No. of reflections	2563	2563	15929	7937
No. of parameters	204	362	1055	574
No. of restraints	6	24	5	13
H-atom treatment	H atoms treated by a mixture of independent and constrained refinement	All H-atom parameters refined	All H-atom parameters constrained	H atoms treated by a mixture of independent and constrained refinement
Δρ_max_, Δρ_min_ (e Å^−3^)	0.23, −0.15	0.19, −0.11	0.55, −0.39	0.24, −0.16
Absolute structure	Flack *x* determined using 1015 quotients [(*I*^+^) − (*I*^−^)]/[(*I*^+^) + (*I*^−^)] (Parsons *et al.*, 2013 ▸)	Hooft *et al.* (2010 ▸)	Flack *x* determined using 6275 quotients [(*I*^+^) − (*I*^−^)]/[(*I*^+^) + (*I*^−^)] (Parsons *et al.*, 2013 ▸)	3363 of Friedel pairs used in the refinement
Absolute structure parameter	0.02 (3)	0.03 (2)	0.03 (6)	0.03 (8)

For all determinations: C_11_H_15_NO_4_·2H_2_O. Experiments were carried out at 100 K using a Bruker D8 VENTURE diffractometer. Absorption was corrected for by multi-scan methods (*SADABS*; Krause *et al.*, 2015 ▸). Computer programs: *APEX3* (Bruker, 2019 ▸), *SAINT-Plus* (Bruker, 2019 ▸, 2020 ▸), *SHELXT* (Sheldrick, 2015*a* ▸), *SHELXL* (Sheldrick, 2015*b* ▸), *OLEX2* (Dolomanov *et al.*, 2009 ▸), and *JANA2020* (Petříček *et al.*, 2023 ▸).

**Table 2:** Average intensities of the main and satellite reflections for I·2H_2_O(mod) with respect to the supercell index *m*

Miller index	<*I*>	<*I*/σ>
*m* (main reflection)	238.81	33.96
*m* − 1 (satellite reflection)	16.22	12.06
*m* + 1 (satellite reflection)	16.12	12.09

**Table 3:** Occupancy factors of the water mol­ecules at the six symmetry-independent sites in the supercell corresponding to the disordered water mol­ecule (O6/O7) from the average structure

Site	No suffix	*A*	*B*	*C*	*D*	*E*	Average
O6	0.654 (5)	0.248 (6)	1	0.654 (5)	0.254 (6)	1	0.635
O7	0.346 (5)	0.752 (6)	0	0.346 (5)	0.746 (6)	0	0.365

## References

[bb1] Allen, F. H. (2002). *Acta Cryst.* B**58**, 380–388. 10.1107/s0108768102003890 12037359

[bb2] Aroyo, M. I., Perez-Mato, J. M., Capillas, C., Kroumova, E., Ivantchev, S., Madariaga, G., Kirov, A. & Wondratschek, H. (2006). *Z. Kristallogr.* **221**, 15–27.

[bb3] Bednarchuk, T. J., Hornfeck, W., Kinzhybalo, V., Zhou, Z., Dušek, M. & Pietraszko, A. (2019). *Acta Cryst.* B**75**, 1144–1151. 10.1107/S2052520619013155 32830694

[bb4] Bourhis, L. J., Dolomanov, O. V., Gildea, R. J., Howard, J. A. K. & Puschmann, H. (2015). *Acta Cryst.* A**71**, 59–75. 10.1107/S2053273314022207 PMC4283469 25537389

[bb5] Brock, C. P. (2016). *Acta Cryst.* B**72**, 807–821. 10.1107/S2052520616017297 27910831

[bb6] Bruffy, S., Meza, A., Soler, J., Doyon, T., Young, S., Lim, J., Huseth, K., Garcia-Borràs, M. & Buller, A. (2023). *ChemRxiv*. doi: 10.26434/chemrxiv-2023-2v3cq. 10.1038/s41557-024-01647-1 PMC11611667 39333392

[bb7] Bruker (2019). *APEX3* and *SAINT-Plus*. Bruker AXS Inc., Madison, Wisconsin, USA.

[bb8] Bruker (2020). *SAINT-Plus*. Bruker AXS Inc., Madison, Wisconsin, USA.

[bb9] Bruno, I. J., Cole, J. C., Kessler, M., Luo, J., Motherwell, W. D. S., Purkis, L. H., Smith, B. R., Taylor, R., Cooper, R. I., Harris, S. E. & Orpen, A. G. (2004). *J. Chem. Inf. Comput. Sci.* **44**, 2133–2144. 10.1021/ci049780b 15554684

[bb10] Bryndal, I., Picur, B. & Lis, T. (2003). *J. Mol. Struct.* **647**, 295–310.

[bb11] Cepeda, J., Balda, R., Beobide, G., Castillo, O., Fernández, J., Luque, A., Pérez-Yáñez, S. & Román, P. (2012). *Inorg. Chem.* **51**, 7875–7888. 10.1021/ic3009392 22726123

[bb12] Dolomanov, O. V., Bourhis, L. J., Gildea, R. J., Howard, J. A. K. & Puschmann, H. (2009). *J. Appl. Cryst.* **42**, 339–341.

[bb13] Doyon, T. J., Kumar, P., Thein, S., Kim, M., Stitgen, A., Grieger, A. M., Madigan, C., Willoughby, P. H. & Buller, A. R. (2022). *ChemBioChem*, **23**, e202100577. 10.1002/cbic.202100577 PMC8796315 34699683

[bb14] Evain, M., Petricek, V., Coué, V., Dessapt, R., Bujoli-Doeuff, M. & Jobic, S. (2006). *Acta Cryst.* B**62**, 790–797. 10.1107/S0108768106025791 16983160

[bb15] Frisch, M. J., Trucks, G. W., Schlegel, H. B., Scuseria, G. E., Robb, M. A., Cheeseman, J. R., Scalmani, G., Barone, V., Petersson, G. A., Nakatsuji, H., Li, X., Caricato, M., Marenich, A. V., Bloino, J., Janesko, B. G., Gomperts, R., Mennucci, B., Hratchian, H. P., Ortiz, J. V., Izmaylov, A. F., Sonnenberg, J. L., Williams, Ding, F., Lipparini, F., Egidi, F., Goings, J., Peng, B., Petrone, A., Henderson, T., Ranasinghe, D., Zakrzewski, V. G., Gao, J., Rega, N., Zheng, G., Liang, W., Hada, M., Ehara, M., Toyota, K., Fukuda, R., Hasegawa, J., Ishida, M., Nakajima, T., Honda, Y., Kitao, O., Nakai, H., Vreven, T., Throssell, K., Montgomery Jr., J. A., Peralta, J. E., Ogliaro, F., Bearpark, M. J., Heyd, J. J., Brothers, E. N., Kudin, K. N., Staroverov, V. N., Keith, T. A., Kobayashi, R., Normand, J., Raghavachari, K., Rendell, A. P., Burant, J. C., Iyengar, S. S., Tomasi, J., Cossi, M., Millam, J. M., Klene, M., Adamo, C., Cammi, R., Ochterski, J. W., Martin, R. L., Morokuma, K., Farkas, O., Foresman, J. B. & Fox, D. J. (2016). *GAUSSIAN16*. Revision C.01. Gaussian Inc., Wallingford, CT, USA. https://gaussian.com/.

[bb16] Gil-García, R., Madariaga, G., Jiménez-Pérez, A., Herrán-Torres, I., Gago-González, A., Ugalde, M., Januskaitis, V., Barrera-García, J., Insausti, M. S., Galletero, M., Borrás, J., Cuevas, J. V., Pedrido, R., Gómez-Saiz, P., Lezama, L. & García-Tojal, J. (2023). *Cryst­EngComm*, **25**, 2213–2226.

[bb17] Groom, C. R. & Allen, F. H. (2014). *Angew. Chem. Int. Ed.* **53**, 662–671. 10.1002/anie.201306438 24382699

[bb18] Groom, C. R., Bruno, I. J., Lightfoot, M. P. & Ward, S. C. (2016). *Acta Cryst.* B**72**, 171–179. 10.1107/S2052520616003954 PMC4822653 27048719

[bb19] Guzei, I. A. (2014). *J. Appl. Cryst.* **47**, 806–809.

[bb20] Hernandez, K., Zelen, I., Petrillo, G., Usón, I., Wandtke, C. M., Bujons, J., Joglar, J., Parella, T. & Clapés, P. (2015). *Angew. Chem. Int. Ed.* **54**, 3013–3017. 10.1002/anie.201411484 25611820

[bb21] Hooft, R. W. W., Straver, L. H. & Spek, A. L. (2010). *J. Appl. Cryst.* **43**, 665–668. 10.1107/S0021889807059870 PMC2467520 19461838

[bb22] Janner, A. & Janssen, T. (1977). *Phys. Rev. B*, **15**, 643–658.

[bb23] Janssen, T. (2012). *Acta Cryst.* A**68**, 667–674. 10.1107/S0108767312033715 23075609

[bb24] Kleemiss, F., Dolomanov, O. V., Bodensteiner, M., Peyerimhoff, N., Midgley, L., Bourhis, L. J., Genoni, A., Malaspina, L. A., Jayatilaka, D., Spencer, J. L., White, F., Grundkötter-Stock, B., Steinhauer, S., Lentz, D., Puschmann, H. & Grabowsky, S. (2021). *Chem. Sci.* **12**, 1675–1692. 10.1039/d0sc05526c PMC8179328 34163928

[bb25] Kozuch, S. & Martin, J. M. L. (2012). *ACS Catal.* **2**, 2787–2794.

[bb26] Krause, L., Herbst-Irmer, R., Sheldrick, G. M. & Stalke, D. (2015). *J. Appl. Cryst.* **48**, 3–10. 10.1107/S1600576714022985 PMC4453166 26089746

[bb27] Kumar, P., Meza, A., Ellis, J. M., Carlson, G. A., Bingman, C. A. & Buller, A. R. (2021). *ACS Chem. Biol.* **16**, 86–95. 10.1021/acschembio.0c00753 PMC8331687 33337128

[bb28] Neese, F. (2012). *WIREs Comput. Mol. Sci.* **2**, 73–78.

[bb29] Neese, F. (2018). *WIREs Comput. Mol. Sci.* **8**, e1327.

[bb30] Palatinus, L. & Chapuis, G. (2007). *J. Appl. Cryst.* **40**, 786–790.

[bb31] Parsons, S., Flack, H. D. & Wagner, T. (2013). *Acta Cryst.* B**69**, 249–259. 10.1107/S2052519213010014 PMC3661305 23719469

[bb32] Petříček, V., Dušek, M. & Palatinus, L. (2014). *Z. Kristallogr.* **229**, 345–352.

[bb33] Petříček, V., Eigner, V., Dušek, M. & Čejchan, A. (2016). *Z. Kristallogr.* **231**, 301–312.

[bb34] Petříček, V., Palatinus, L., Plášil, J. & Dušek, M. (2023). *Z. Kristallogr.* **238**, 271–282.

[bb35] Pinheiro, C. B. & Abakumov, A. M. (2015). *IUCrJ*, **2**, 137–154. 10.1107/S2052252514023550 PMC4285887 25610634

[bb36] Rekis, T., Schaller, A. M., Kotla, S. R., Schönleber, A., Noohinejad, L., Tolkiehn, M., Paulmann, C. & van Smaalen, S. (2021). *IUCrJ*, **8**, 139–147. 10.1107/S2052252520015912 PMC7792995 33520250

[bb37] Rekis, T., Schönleber, A. & van Smaalen, S. (2020). *Acta Cryst.* B**76**, 18–27. 10.1107/S2052520619014938 PMC8788847 32831236

[bb38] Savic, V., Eder, F., Göb, C., Mihovilovic, M. D., Stanetty, C. & Stöger, B. (2021). *Acta Cryst.* B**77**, 83–92.

[bb39] Schaffer, J. E., Reck, M. R., Prasad, N. K. & Wencewicz, T. A. (2017). *Nat. Chem. Biol.* **13**, 737–744. 10.1038/nchembio.2374 28504677

[bb40] Schönleber, A. (2011). *Z. Kristallogr.* **226**, 499–517.

[bb41] Schönleber, A. (2023). *Phys. Sci. Rev.* **2023**, https://doi.org/10.1515/psr-2018-0140.

[bb42] Scott, T. A., Heine, D., Qin, Z. & Wilkinson, B. (2017). *Nat. Commun.* **8**, 15935. 10.1038/ncomms15935 PMC5490192 28649989

[bb43] Sheldrick, G. M. (2015*a*). *Acta Cryst.* A**71**, 3–8.

[bb44] Sheldrick, G. M. (2015*b*). *Acta Cryst.* C**71**, 3–8.

[bb45] Smaalen, S. van (1995). *Crystallogr. Rev.* **4**, 79–202.

[bb46] Smaalen, S. van (2004). *Z. Kristallogr.* **219**, 681–691.

[bb47] Spek, A. L. (2020). *Acta Cryst.* E**76**, 1–11. 10.1107/S2056989019016244 PMC6944088 31921444

[bb48] Wagner, T. & Schönleber, A. (2009). *Acta Cryst.* B**65**, 249–268. 10.1107/S0108768109015614 19461136

[bb49] Wolff, P. M. de (1974). *Acta Cryst.* A**30**, 777–785.

[bb50] Wolff, P. M. de (1977). *Acta Cryst.* A**33**, 493–497.

[bb51] Yamamoto, A. (1996). *Acta Cryst.* A**52**, 509–560.

