# Supplementary material for: Occupational modulation in the (3+1)-dimensional incommensurate structure of (2S,3S)-2-amino-3-hy­droxy-3-methyl-4-phen­oxy­butanoic acid dihydrate
Source: Acta Crystallogr C Struct Chem. 2024 Aug 8;80(Pt 9):523–33. doi: 10.1107/S2053229624007009 (PMC11371000; doi:10.1107/S2053229624007009)
Supplement: Supplementary file 6 [file c-80-00523-sup6.pdf]

Acta Cryst C STRUCTURAL CHEMISTRY

Volume 80 (2024)

Supporting information for article:

Occupational modulation in the (3+1)-dimensional incommensurate structure of (2*S*,3*S*)-2-amino-3-hydroxy-3-methyl-4-phenoxy-butanoic acid dihydrate

Kyana M. Sanders, Samantha K. Bruffy, Andrew R. Buller, Václav Petříček and Ilia A. Guzei

S1. Experimental

S1.1. Additional Superspace Refinement Details

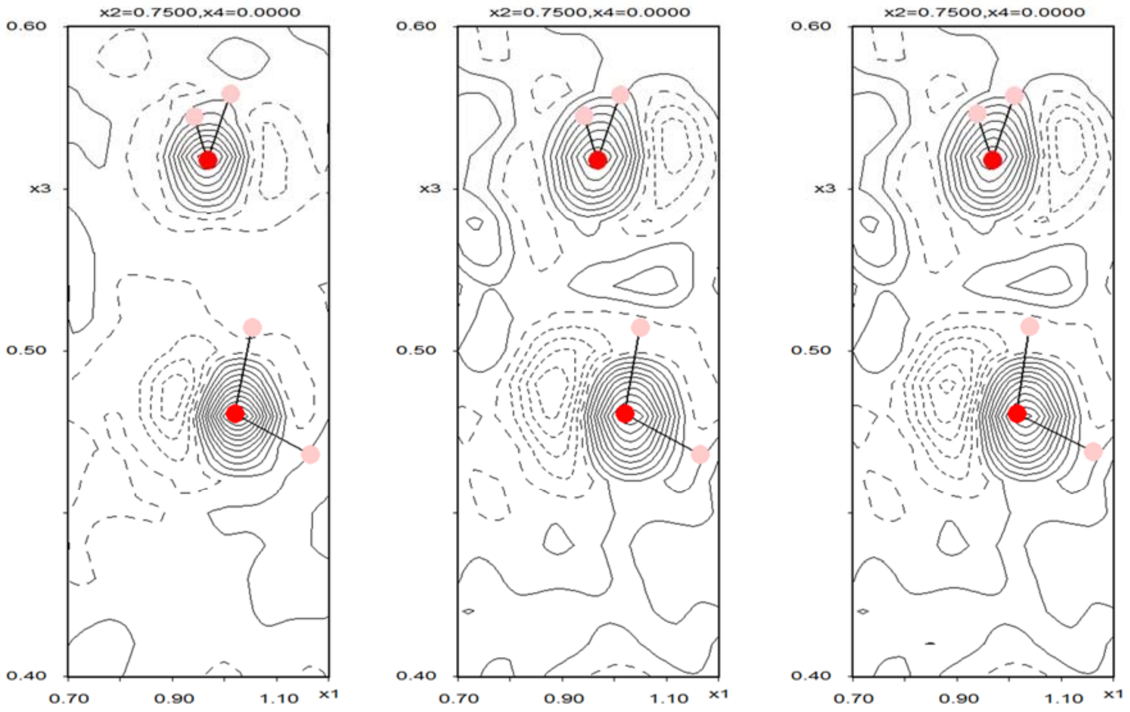

Figure S1 Electron density plots showing the (3+1)D modulation of atoms O5, O6 and O7 in the $x1$-$x3$ plane as a function of $x4$, with the electron density summed over the interval of $0.65 < x2 < 0.85$. The motion of the atoms described using 2 harmonics (left), 1 harmonic (middle), and a crenel (right) function are shown.

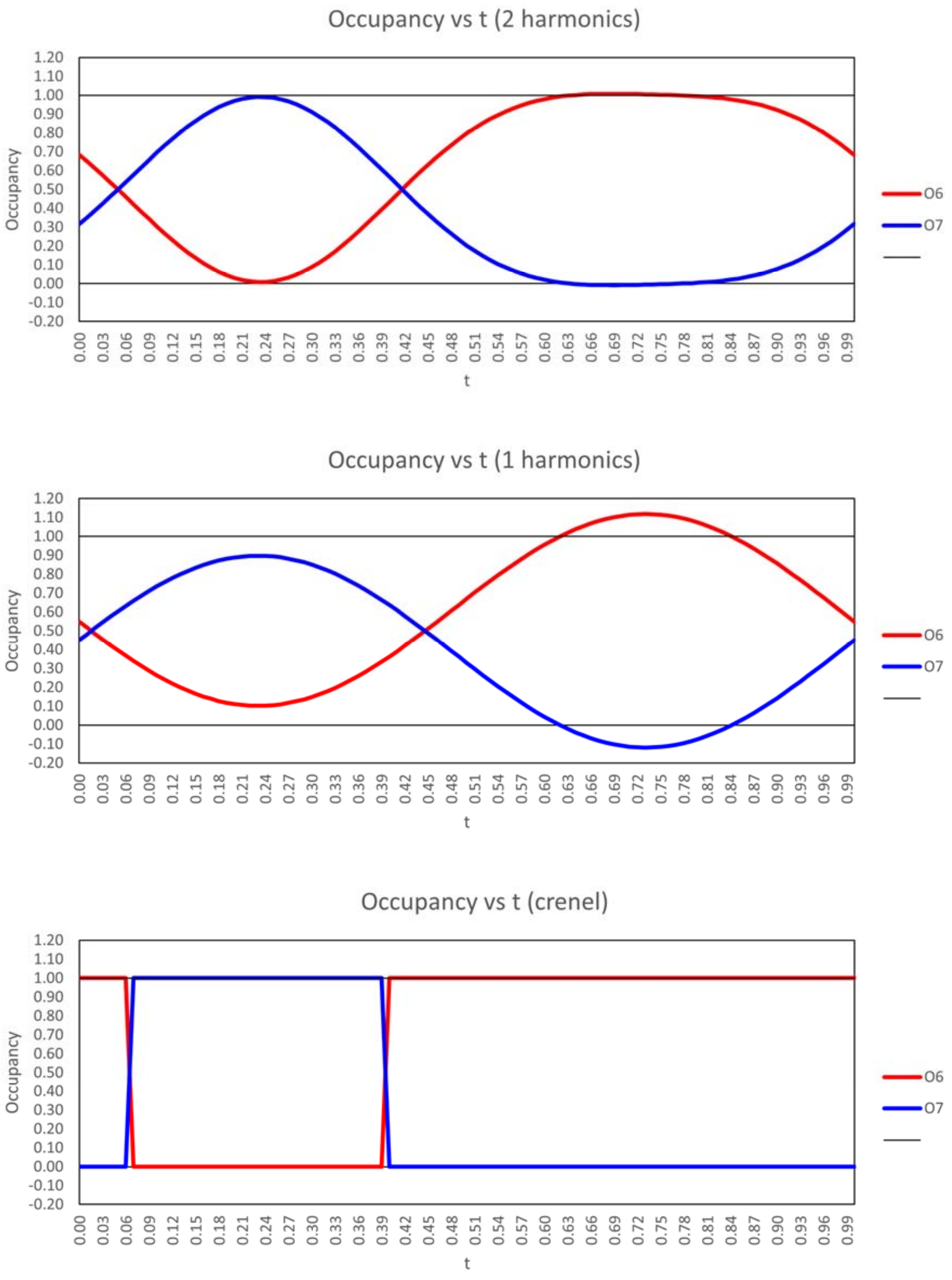

Figure S2 Plots of the 2 harmonic, 1 harmonic, and crenel functions used to model the occupancy modulations of atoms O6 and O7 vs t.

S1.2. Density Functional Theory (DFT) Calculations

Table S1 Optimized atomic coordinates for symmetry-distinct sites in **I**-DFT.

Atom	Atomic No.	x	y	z
O1	8	1.952717	-0.170840	-1.044409
O2	8	1.692860	-1.538724	-2.826000
O3	8	-1.889691	-2.339260	-0.281071
O4	8	-1.284287	-0.045420	1.026666
N5	7	-0.858717	-1.711437	-2.682737
C6	6	1.300488	-0.895945	-1.817339
C7	6	-0.220769	-1.054040	-1.489666
C8	6	-0.516195	-1.917742	-0.220624
C9	6	0.323573	-3.191713	-0.160023
C10	6	-0.295409	-1.080079	1.042053
C11	6	-1.369375	0.820087	2.087047
C12	6	-0.508393	0.796790	3.186560
C13	6	-0.683954	1.731021	4.211678
C14	6	-1.700992	2.679238	4.149671
C15	6	-2.555909	2.692382	3.043494
C16	6	-2.395216	1.770110	2.016166
H17	1	-0.677279	-0.074700	-1.361841
H18	1	-0.064639	-2.133830	-3.203261
H19	1	-1.313759	-1.035589	-3.296071
H20	1	-1.560536	-2.399029	-2.390647
H21	1	0.173999	-3.806557	-1.050096
H22	1	1.385925	-2.957141	-0.079985
H23	1	0.026816	-3.781140	0.708808
H24	1	-2.440679	-1.588864	-0.013887
H25	1	-0.434891	-1.716535	1.920938
H26	1	0.710300	-0.654795	1.037545
H27	1	0.291131	0.071819	3.256482
H28	1	-0.012417	1.708557	5.062604
H29	1	-1.828648	3.399232	4.949171
H30	1	-3.352854	3.424764	2.980323
H31	1	-3.052136	1.770703	1.154258

S2. Additional Plots for I·2H_2O(mod) Discussion

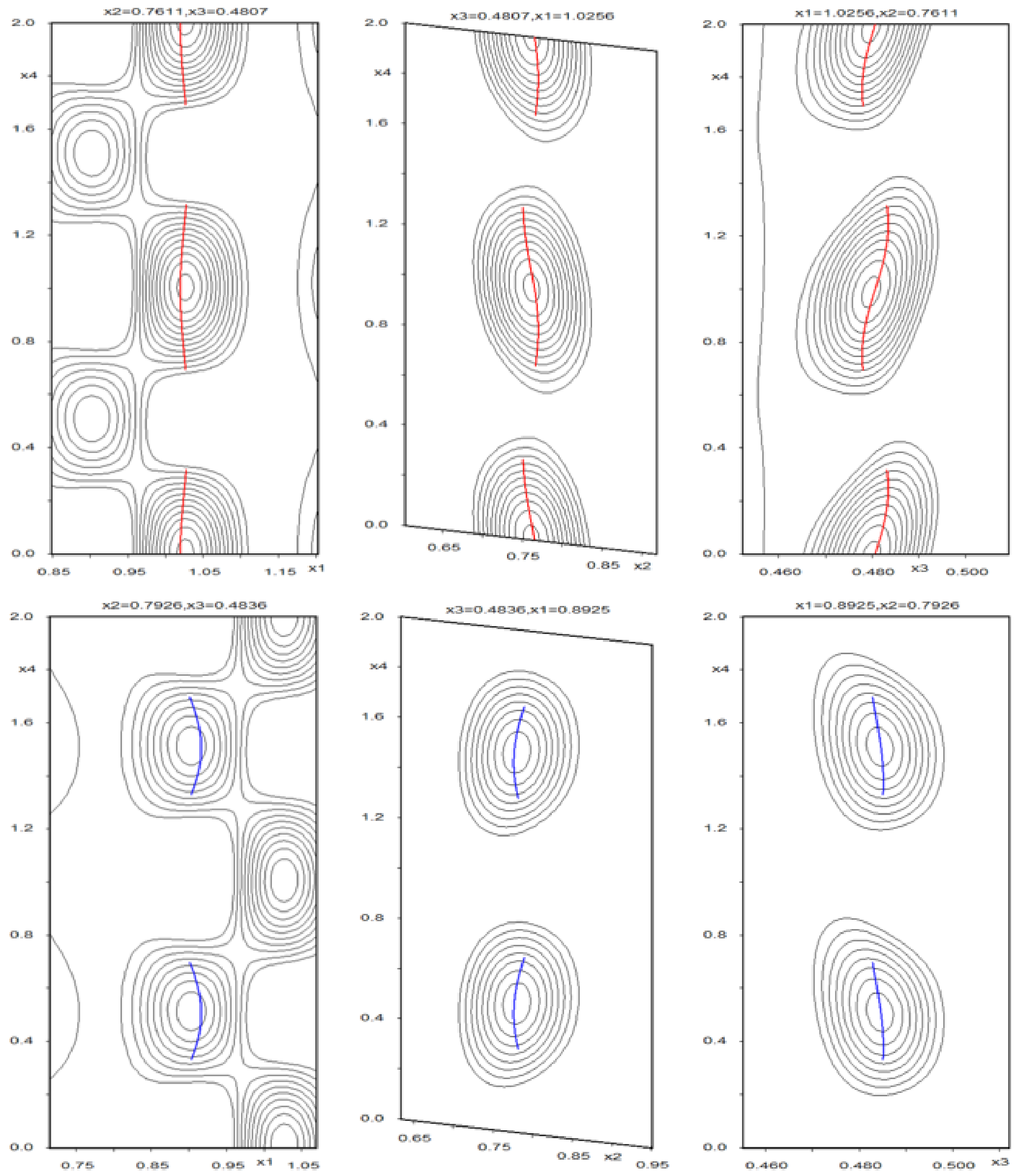

Figure S3 Electron density plots showing the (3+1)D modulation of the O6 and O7 atoms along *x*1 (left), *x*2 (middle), and x3 (right) as a function of *x*4 in the final superspace model using two harmonic waves for the occupancy modulation. For all plots, the electron density is summed over a thickness of 1 Å in the remaining directions. Solid and dashed black lines represent areas of positive and negative electron density. This occupational modulation manifests as positional disorder (right) where the water molecule is split over the O6 (red, top) and O7 (blue, bottom) sites which have an occupancy ratio of 62.4(3):37.6(3). The curves of the red and blue lines represent the paths of motion of the O6 and O7 atoms along the primary axes *x*1 (left), *x*2 (middle), and x3 (right) as a function of *x*4.

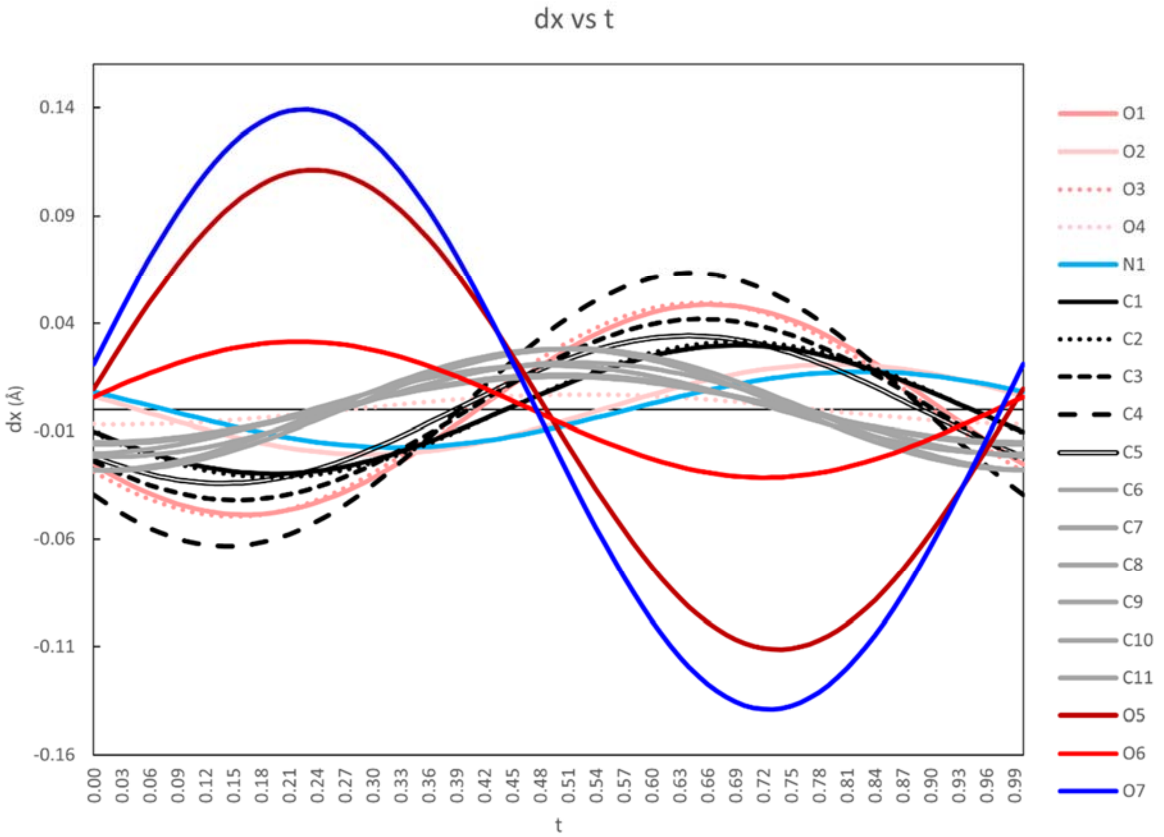
dx vs t
dx (Å)
t
O1
O2
O3
O4
N1
C1
C2
C3
C4
C5
C6
C7
C8
C9
C10
C11
O5
O6
O7

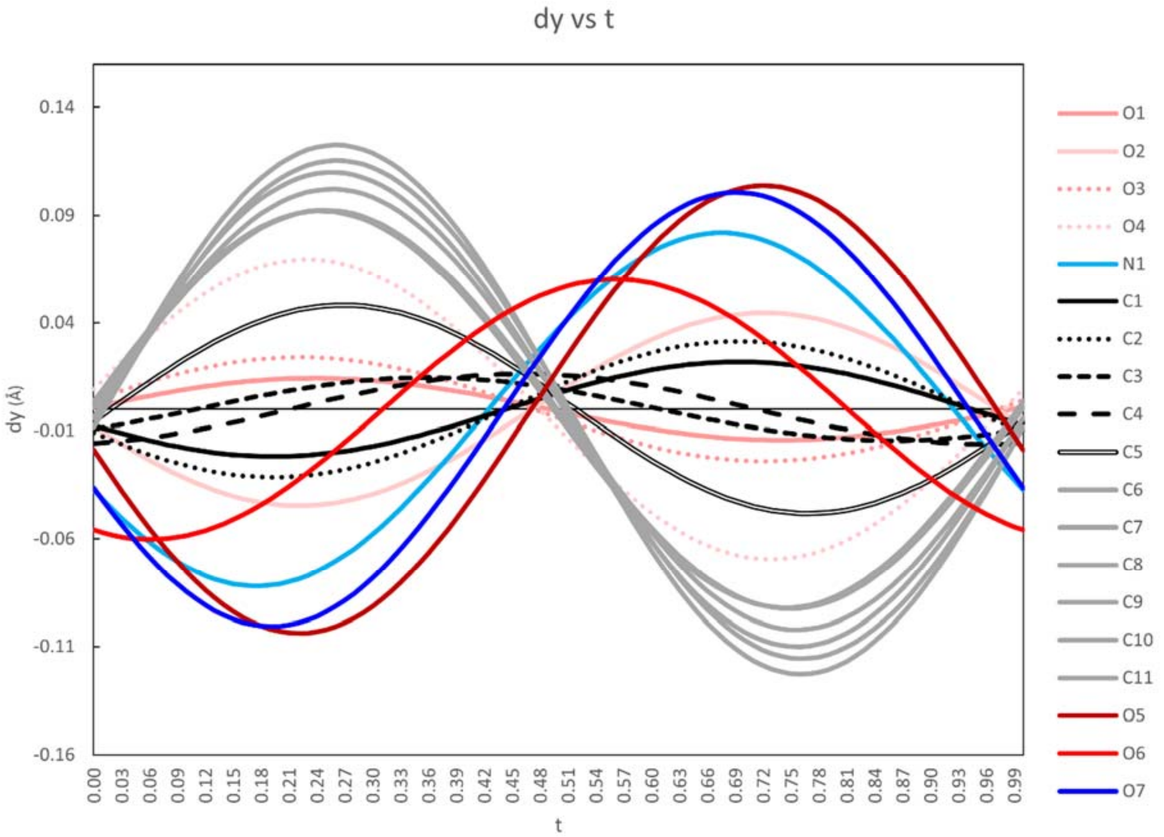
dy vs t
dy (Å)
t
O1
O2
O3
O4
N1
C1
C2
C3
C4
C5
C6
C7
C8
C9
C10
C11
O5
O6
O7

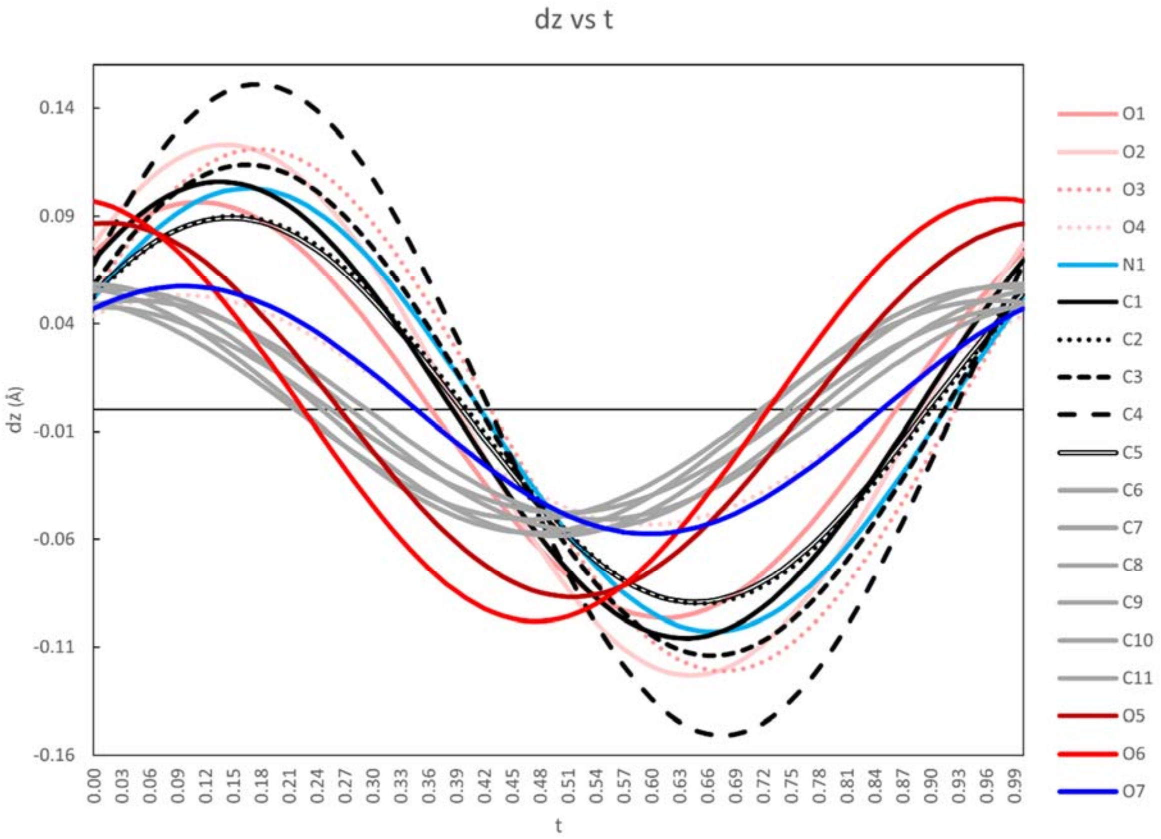

Figure S4 Plots of dx, dy, dz vs t for all non-H atoms in the final superspace model using two harmonic waves for the occupancy modulation.

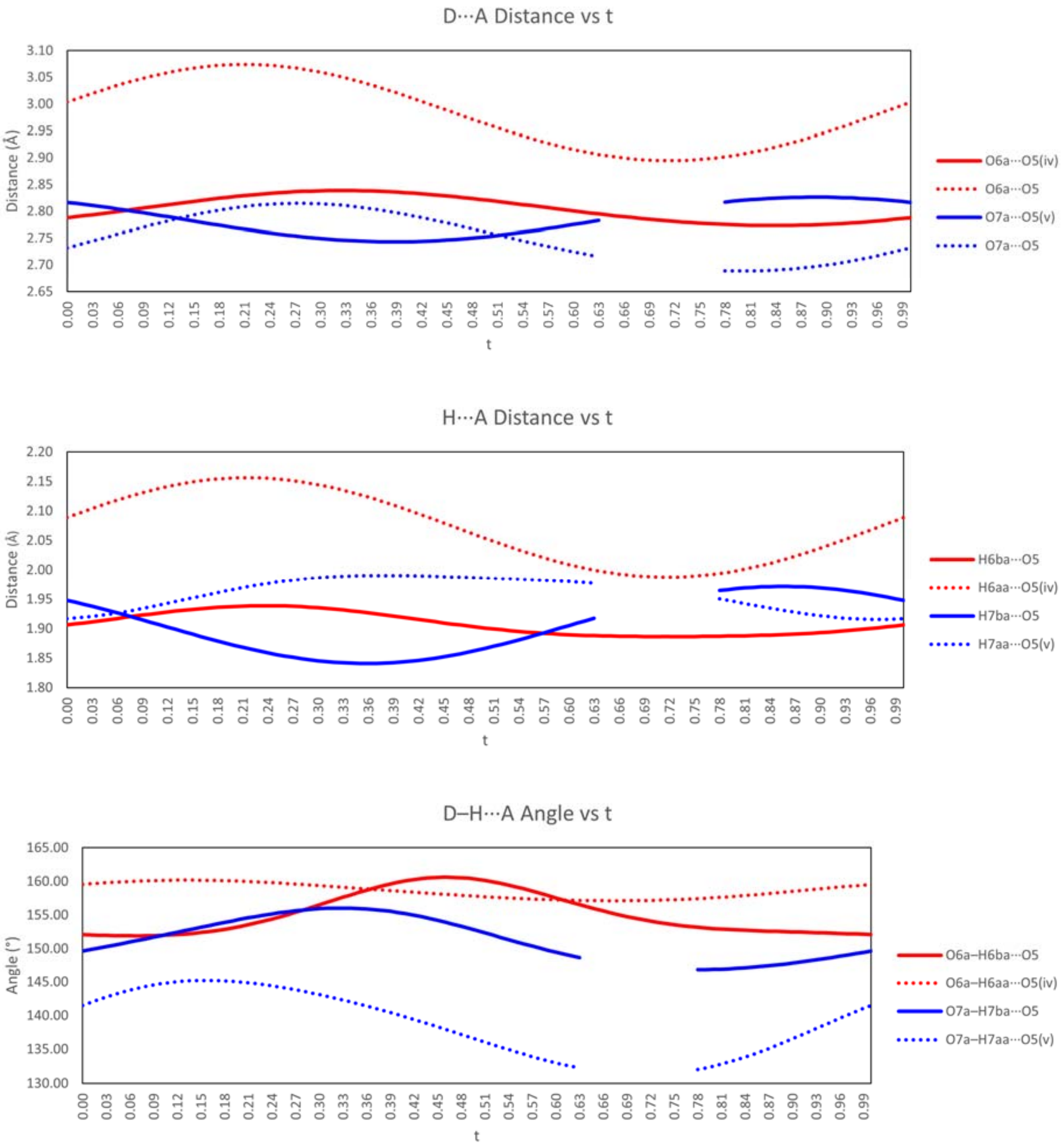

Figure S5 Plots of H-bonding interactions where atoms O6a and O7a serve as H-donors vs t in the final superspace model using two harmonic waves for the occupancy modulation. Symmetry code(s): (i) x−1, y, z; (ii) x−1/2, −y+1/2, −z+1; (iii) x+1, y+1, z; (iv) x+1/2, −y+3/2, −z+1; (v) x−1/2, −y+3/2, −z+1.

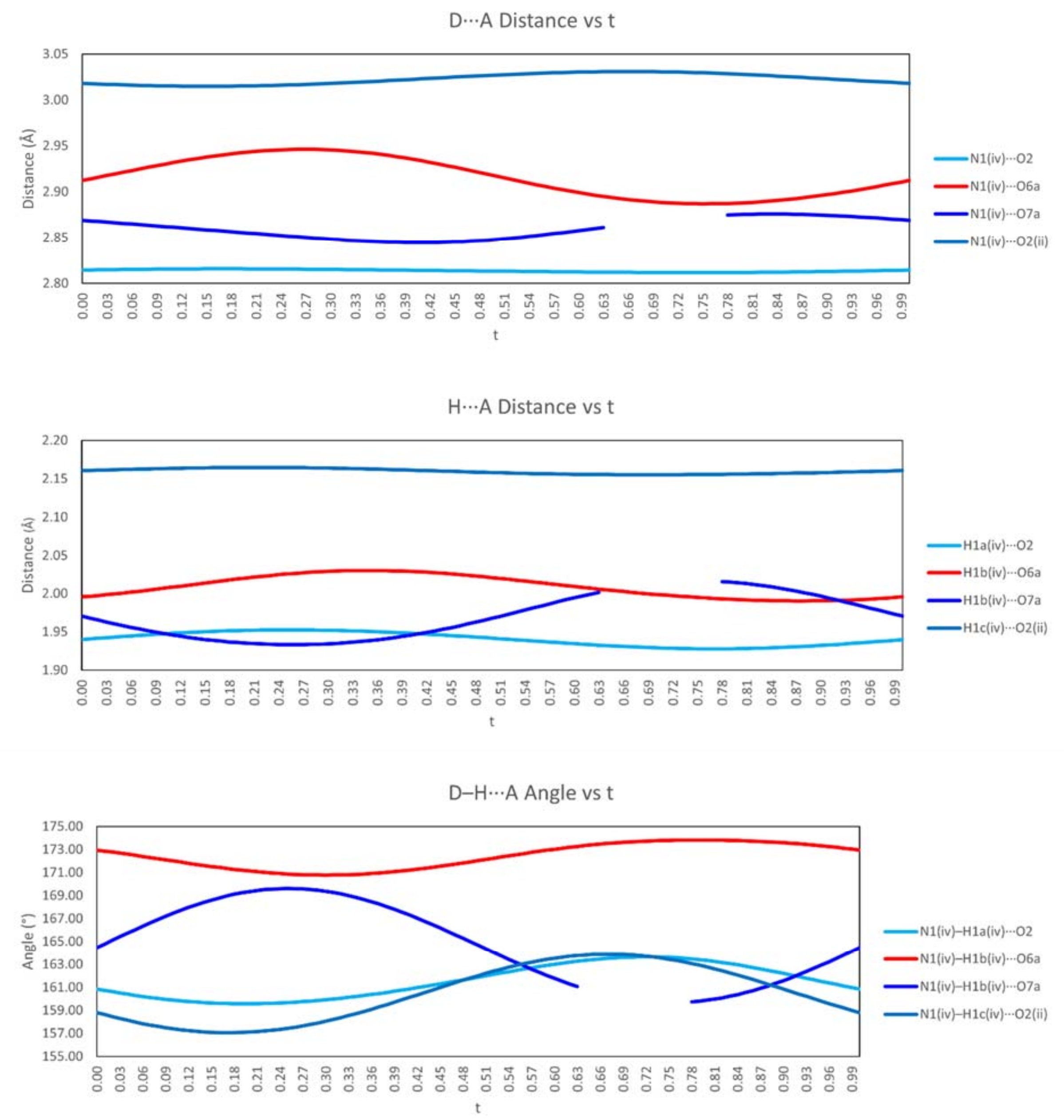

Figure S6 Plots of H-bonding interactions where atom N1^{iv} serves as an H-donor vs t in the final superspace model using two harmonic waves for the occupancy modulation. Symmetry code(s): (i) x−1, y, z; (ii) x−1/2, −y+1/2, −z+1; (iii) x+1, y+1, z; (iv) x+1/2, −y+3/2, −z+1; (v) x−1/2, −y+3/2, −z+1.

S3. Geometric Parameters for I·2H2O(av), I·2H2O(NS2), I·2H2O(supercell), and I·2H2O(mod).

Table S2 Selected hydrogen-bond parameters for **I**·2H2O(av).

D—H···*A*	*D*—H (Å)	H···*A* (Å)	*D*···*A* (Å)	*D*—H···*A* (°)
O3—H3···O1^{i}	0.84 (2)	1.94 (2)	2.7398 (16)	159 (2)
N1—H1A···O2^{ii}	0.91 (2)	1.94 (2)	2.8134 (18)	158 (2)
N1—H1B···O2^{i}	0.88 (2)	2.17 (2)	3.0237 (18)	162.6 (19)
N1—H1C···O6^{ii}	0.93 (2)	2.00 (2)	2.919 (3)	173 (2)
N1—H1C···O7^{ii}	0.93 (2)	1.91 (2)	2.821 (5)	167 (2)
O5—H5C···O1	0.91 (3)	1.82 (3)	2.7071 (17)	166 (2)
O5—H5D···O3^{iii}	0.89 (3)	1.93 (3)	2.7937 (17)	164 (2)
O6—H6A···O5^{iv}	0.958 (4)	2.089 (19)	2.980 (7)	154 (3)
O6—H6B···O5	0.958 (4)	1.877 (11)	2.800 (3)	161 (3)
O7—H7A···O5^{v}	0.958 (4)	2.06 (4)	2.849 (12)	139 (5)
O7—H7B···O5	0.958 (4)	1.82 (2)	2.726 (4)	157 (5)

Symmetry code(s): (i) x−1, y, z; (ii) x−1/2, −y+1/2, −z+1; (iii) x+1, y+1, z; (iv) x+1/2, −y+3/2, −z+1; (v) x−1/2, −y+3/2, −z+1.

Table S3 Selected hydrogen-bond parameters for **I**·2H2O(NS2).

D—H···*A*	*D*—H (Å)	H···*A* (Å)	*D*···*A* (Å)	*D*—H···*A* (°)
O3—H3···O1^{i}	0.931 (10)	1.850 (10)	2.7357 (7)	158.2 (8)
N1—H1a···O2^{ii}	1.001 (11)	1.866 (11)	2.8176 (8)	157.6 (8)
N1—H1b···O2^{i}	0.986 (11)	2.070 (11)	3.0259 (8)	162.7 (9)
N1—H1c···O6^{ii}	1.036 (9)	1.890 (10)	2.9180 (15)	171.6 (9)
N1—H1c···O7^{ii}	1.036 (9)	1.801 (10)	2.816 (2)	165.5 (8)
O5—H5c···O1	0.986 (12)	1.733 (12)	2.7060 (8)	168.4 (10)
O5—H5d···O3^{iii}	0.961 (10)	1.847 (10)	2.7933 (8)	167.7 (9)
O6—H6a···O5^{iv}	0.959 (3)	2.061 (8)	2.987 (4)	161.8 (18)
O6—H6b···O5	0.959 (3)	1.872 (5)	2.8044 (14)	163.5 (12)
O7—H7a···O5^{v}	0.959 (3)	1.99 (2)	2.846 (6)	147 (3)
O7—H7b···O5	0.959 (3)	1.841 (13)	2.7264 (18)	152 (2)

Symmetry code(s): (i) x−1, y, z; (ii) x−1/2, −y+1/2, −z+1; (iii) x+1, y+1, z; (iv) x+1/2, −y+3/2, −z+1; (v) x−1/2, −y+3/2, −z+1.

Table S4 Selected hydrogen-bond parameters for **I**·2H2O(supercell).

D—H···*A*	*D*—H (Å)	H···*A* (Å)	*D*···*A* (Å)	*D*—H···*A* (°)
O3—H3···O1^{i}	0.84	1.92	2.737 (3)	163.9
N1—H1A···O2C^{i}	0.91	1.95	2.816 (3)	157.3
N1—H1B···O2^{i}	0.91	2.17	3.033 (3)	158.5
N1—H1C···O6B^{ii}	0.91	2.00	2.901 (3)	168.8
O3A—H3A···O1A^{i}	0.84	1.93	2.752 (3)	164.0
N1A—H1AA···O2D^{i}	0.91	1.94	2.813 (3)	159.4

N1A—H1AB···O2A^i	0.91	2.17	3.034 (3)	157.5
N1A—H1AC···O6	0.91	2.03	2.931 (4)	171.5
N1A—H1AC···O7	0.91	1.92	2.802 (6)	161.7
O3B—H3B···O1B^i	0.84	1.94	2.749 (3)	160.8
N1B—H1BA···O2E^i	0.91	1.97	2.818 (3)	154.4
N1B—H1BB···O2B^i	0.91	2.15	3.023 (3)	159.4
N1B—H1BC···O6A	0.91	2.03	2.931 (10)	168.2
N1B—H1BC···O7A	0.91	1.97	2.860 (4)	167.1
O3C—H3C···O1C^i	0.84	1.91	2.737 (3)	169.4
N1C—H1CA···O2C^i	0.91	2.17	3.023 (3)	156.0
N1C—H1CB···O6C	0.91	2.01	2.916 (4)	173.9
N1C—H1CB···O7C	0.91	1.94	2.822 (6)	162.6
N1C—H1CC···O2	0.91	1.96	2.820 (3)	155.9
O3D—H3D···O1D^i	0.84	1.92	2.751 (3)	168.6
N1D—H1DA···O2D^i	0.91	2.17	3.024 (3)	156.0
N1D—H1DB···O6D	0.91	2.08	2.988 (10)	173.9
N1D—H1DB···O7D	0.91	1.96	2.847 (4)	164.8
N1D—H1DC···O2A	0.91	1.96	2.816 (3)	157.2
O3E—H3E···O1E^i	0.84	1.92	2.741 (3)	167.2
N1E—H1EA···O2E^i	0.91	2.18	3.034 (3)	156.3
N1E—H1EB···O6E	0.91	2.00	2.901 (3)	169.6
N1E—H1EC···O2B	0.91	1.94	2.814 (3)	159.9
O5—H5C···O1	0.87	1.87	2.705 (3)	160.3
O5—H5D···O3A^iii	0.87	1.95	2.806 (3)	169.2
O5A—H5AC···O1A	0.87	1.85	2.707 (3)	168.5
O5A—H5AD···O3B^iii	0.87	1.94	2.792 (3)	166.1
O5B—H5BC···O3^iv	0.87	1.97	2.812 (3)	161.0
O5B—H5BD···O1B	0.87	1.89	2.712 (3)	156.5
O5C—H5CC···O3C	0.87	1.94	2.794 (3)	166.5
O5C—H5CD···O1D^i	0.87	1.91	2.713 (3)	153.1
O5D—H5DC···O1E^i	0.87	1.86	2.707 (3)	162.2

O5D—H5DD···O3D	0.87	1.95	2.796 (3)	163.4
O5E—H5EC···O1C^{v}	0.87	1.94	2.716 (3)	148.3
O5E—H5ED···O3E	0.87	1.97	2.816 (3)	162.8
O6—H6A···O5	0.87	2.16	2.980 (4)	155.6
O6—H6B···O5C	0.87	2.00	2.829 (4)	158.2
O6A—H6AA···O5D	0.87	1.87	2.701 (10)	159.6
O6A—H6AB···O5A	0.87	2.25	3.099 (11)	163.6
O6B—H6BA···O5E	0.87	1.99	2.797 (3)	154.1
O6B—H6BB···O5B	0.87	2.07	2.914 (3)	164.2
O6C—H6CA···O5C^{iii}	0.87	2.24	3.011 (5)	146.9
O6C—H6CB···O5	0.87	1.99	2.781 (4)	149.9
O6D—H6DA···O5A	0.87	2.34	2.962 (10)	128.3
O6D—H6DB···O5D^{iii}	0.87	2.42	2.981 (12)	122.1
O6E—H6EA···O5E^{iii}	0.87	2.12	2.919 (3)	152.6
O6E—H6EB···O5B	0.87	1.96	2.784 (3)	158.5
O7—H7F···O5^{i}	0.87	2.18	2.857 (7)	134.3
O7—H7G···O5C	0.87	1.97	2.697 (6)	140.5
O7A—H7AA···O5A^{i}	0.87	2.03	2.776 (4)	142.4
O7A—H7AB···O5D	0.87	2.06	2.806 (4)	143.8
O7C—H7CA···O5	0.87	1.95	2.767 (7)	155.5
O7C—H7CB···O5C	0.87	1.98	2.811 (7)	158.5
O7D—H7DA···O5A	0.87	1.94	2.731 (4)	150.4
O7D—H7DB···O5D	0.87	2.05	2.820 (4)	147.2

Symmetry code(s): (i) $x-1, y, z$; (ii) $x, y-1, z$; (iii) $x+1, y, z$; (iv) $x+1, y+1, z$; (v) $x-1, y+1, z$.

Table S5 Selected hydrogen-bond parameters for **I**·2H2O(mod).

D—H···*A*	*D*—H (Å)	H···*A* (Å)	*D*···*A* (Å)	*D*—H···*A* (°)
O3—H3···O1^{i}				
Min.	0.835(13)	1.937(15)	2.7311(18)	153.1(16)
Max.	0.863(13)	1.942(15)	2.7489(18)	161.5(15)
Avg.	0.848(13)	1.939(15)	2.7400(18)	157.4(16)
N1—H1A···O2^{ii}				
Min.	0.901(13)	1.928(12)	2.8118(17)	159.6(15)

Max.	0.911(13)	1.953(12)	2.8160(17)	163.7(15)
Avg.	0.906(13)	1.941(12)	2.8139(17)	161.5(15)
N1—H1C···O2^{i}				
Min.	0.900(11)	2.155(11)	3.015(3)	157.1(14)
Max.	0.901(11)	2.165(11)	3.031(3)	163.9(14)
Avg.	0.901(11)	2.160(11)	3.023(3)	160.4(14)
N1—H1B···O6A^{ii}				
Min.	0.893(12)	1.990(13)	2.887(3)	170.8(14)
Max.	0.931(12)	2.030(13)	2.946(3)	173.8(14)
Avg.	0.912(12)	2.009(13)	2.916(3)	172.4(14)
N1—H1B···O7A^{ii}				
Min.	0.893(12)	1.933(14)	2.845(9)	159.7(15)
Max.	0.931(12)	2.016(14)	2.876(8)	169.6(15)
Avg.	0.915(12)	1.966(14)	2.859(9)	165.3(15)
O5—H5C···O1				
Min.	0.946(13)	1.740(14)	2.6957(18)	157.2(18)
Max.	0.968(15)	1.793(14)	2.7134(18)	175.7(13)
Avg.	0.958(13)	1.767(14)	2.7050(18)	167.2(16)
O5—H5D···O3^{iii}				
Min.	0.952(11)	1.853(12)	2.7835(18)	161.0(9)
Max.	0.963(12)	1.887(11)	2.8177(17)	165.8(12)
Avg.	0.958(11)	1.869(12)	2.7992(18)	163.1(12)
O6A—H6AA···O5^{iv}				
Min.	0.956(18)	1.99(2)	2.895(5)	157(4)
Max.	0.96(2)	2.16(3)	3.074(5)	160(4)
Avg.	0.96(2)	2.07(2)	2.983(4)	159(4)
O6A—H6BA···O5				
Min.	0.955(7)	1.887(13)	2.773(3)	152(3)
Max.	0.960(10)	1.939(15)	2.839(3)	161(3)
Avg.	0.957(9)	1.908(12)	2.805(3)	155(3)
O7A—H7AA···O5^{v}				
Min.	0.96(6)	1.92(6)	2.69(2)	132(8)
Max.	0.96(7)	1.99(7)	2.81(2)	145(8)
Avg.	0.96(6)	1.96(7)	2.757(19)	140(7)
O7A—H7BA···O5				
Min.	0.96(2)	1.84(3)	2.743(12)	147(8)

Max.	0.96(3)	1.97(4)	2.826(12)	156(7)
Avg.	0.96(3)	1.90(4)	2.782(13)	152(7)

Symmetry code(s): (i) x−1, y, z; (ii) x−1/2, −y+1/2, −z+1; (iii) x+1, y+1, z; (iv) x+1/2, −y+3/2, −z+1; (v) x−1/2, −y+3/2, −z+1.

Table S6 Selected bond distances for **I**·2H2O(av), **I**·2H2O(NS2) and **I**·2H2O(mod).

	AV	NS2	MOD_{AVG}	MOD_{MIN}	MOD_{MAX}
O1—C1	1.2560 (19)	1.2563 (7)	1.259 (5)	1.257 (5)	1.262 (5)
O2—C1	1.2559 (19)	1.2533 (7)	1.255 (3)	1.254 (4)	1.256 (4)
O3—H3	0.84 (2)	0.931 (10)	0.848 (13)	0.835 (14)	0.863 (14)
O3—C3	1.4306 (18)	1.4255 (7)	1.43 (2)	1.43 (2)	1.43 (2)
O4—C5	1.4270 (18)	1.4226 (7)	1.427 (10)	1.425 (11)	1.428 (11)
O4—C6	1.3696 (18)	1.3625 (7)	1.3704 (14)	1.3688 (14)	1.3718 (14)
N1—H1A	0.91 (2)	1.001 (11)	0.906 (13)	0.901 (13)	0.911 (13)
N1—H1B	0.88 (2)	0.986 (11)	0.912 (12)	0.893 (12)	0.931 (12)
N1—H1C	0.93 (2)	1.036 (9)	0.901 (17)	0.900 (17)	0.901 (17)
N1—C2	1.4930 (18)	1.4920 (7)	1.494 (5)	1.494 (5)	1.495 (5)
C1—C2	1.535 (2)	1.5348 (8)	1.54 (2)	1.53 (2)	1.54 (2)
C2—H2	1	1.081 (7)	1.000 (2)	1.000 (2)	1.000 (2)
C2—C3	1.562 (2)	1.5616 (8)	1.5629 (19)	1.5605 (19)	1.5645 (19)
C3—C4	1.523 (2)	1.5225 (8)	1.523 (7)	1.521 (8)	1.526 (8)
C3—C5	1.527 (2)	1.5268 (8)	1.5297 (16)	1.5281 (16)	1.5317 (16)
C4—H4A	0.98	1.071 (9)	0.980 (2)	0.979 (2)	0.981 (2)
C4—H4B	0.98	1.040 (10)	0.9800 (12)	0.9787 (12)	0.9813 (12)
C4—H4C	0.98	1.066 (9)	0.980 (14)	0.979 (14)	0.981 (14)
C5—H5A	0.99	1.094 (8)	0.9900 (12)	0.9896 (12)	0.9904 (12)
C5—H5B	0.99	1.079 (7)	0.990 (13)	0.990 (13)	0.990 (13)
C6—C7	1.394 (2)	1.3948 (8)	1.394 (11)	1.393 (11)	1.395 (11)
C6—C11	1.394 (2)	1.3960 (8)	1.397 (14)	1.396 (14)	1.397 (14)
C7—H7	0.95	1.069 (9)	0.950 (10)	0.950 (10)	0.950 (10)
C7—C8	1.392 (2)	1.3972 (9)	1.3960 (19)	1.3950 (19)	1.3969 (19)
C8—H8	0.95	1.081 (10)	0.950 (7)	0.950 (7)	0.950 (7)
C8—C9	1.381 (3)	1.3873 (10)	1.385 (14)	1.383 (14)	1.388 (14)
C9—H9	0.95	1.083 (9)	0.9500 (15)	0.9500 (16)	0.9500 (16)
C9—C10	1.394 (2)	1.3999 (10)	1.391 (10)	1.390 (11)	1.392 (11)
C10—H10	0.95	1.089 (8)	0.950 (9)	0.950 (9)	0.950 (9)
C10—C11	1.385 (2)	1.3876 (9)	1.3923 (19)	1.3898 (19)	1.3949 (19)
C11—H11	0.95	1.075 (9)	0.950 (7)	0.950 (7)	0.950 (7)
O5—H5C	0.91 (3)	0.986 (12)	0.958 (13)	0.946 (15)	0.968 (15)
O5—H5D	0.89 (3)	0.961 (10)	0.958 (11)	0.952 (12)	0.963 (12)
O6—H6A	0.958 (4)	0.959 (3)	0.96 (2)	0.96 (3)	0.96 (3)
O6—H6B	0.958 (4)	0.959 (3)	0.957 (9)	0.955 (12)	0.960 (12)
O7—H7A	0.958 (4)	0.959 (3)	0.96 (6)	0.96 (8)	0.96 (8)
O7—H7B	0.958 (4)	0.959 (3)	0.96 (3)	0.96 (3)	0.96 (3)

Table S7 Selected bond distances for **I**·2H2O(supercell).

	No Suffix	A	B	C	D	E
O1—C1	1.259 (3)	1.260 (3)	1.259 (3)	1.261 (3)	1.259 (3)	1.258 (3)
O2—C1	1.255 (3)	1.257 (4)	1.256 (3)	1.258 (3)	1.261 (4)	1.259 (3)
O3—H3	0.84	0.84	0.84	0.84	0.84	0.84
O3—C3	1.438 (3)	1.437 (3)	1.435 (3)	1.435 (3)	1.436 (3)	1.436 (3)
O4—C5	1.430 (3)	1.428 (3)	1.428 (3)	1.429 (3)	1.429 (3)	1.428 (3)
O4—C6	1.370 (3)	1.371 (3)	1.369 (3)	1.373 (3)	1.373 (3)	1.375 (3)
N1—H1A	0.91	0.91	0.91	0.91	0.91	0.91
N1—H1B	0.91	0.91	0.91	0.91	0.91	0.91
N1—H1C	0.91	0.91	0.91	0.91	0.91	0.91
N1—C2	1.497 (3)	1.495 (3)	1.495 (3)	1.494 (3)	1.497 (3)	1.497 (3)
C1—C2	1.540 (3)	1.537 (3)	1.541 (3)	1.538 (3)	1.539 (3)	1.537 (3)
C2—H2	1	1	1	1	1	1
C2—C3	1.564 (3)	1.561 (3)	1.563 (3)	1.572 (3)	1.564 (4)	1.566 (3)
C3—C4	1.528 (3)	1.530 (3)	1.527 (3)	1.525 (3)	1.525 (3)	1.524 (3)
C3—C5	1.527 (3)	1.530 (3)	1.529 (3)	1.530 (3)	1.533 (4)	1.533 (3)
C4—H4A	0.98	0.98	0.98	0.98	0.98	0.98
C4—H4B	0.98	0.98	0.98	0.98	0.98	0.98
C4—H4C	0.98	0.98	0.98	0.98	0.98	0.98
C5—H5A	0.99	0.99	0.99	0.99	0.99	0.99
C5—H5B	0.99	0.99	0.99	0.99	0.99	0.99
C6—C7	1.396 (4)	1.396 (4)	1.397 (4)	1.397 (4)	1.397 (4)	1.395 (4)
C6—C11	1.393 (4)	1.397 (4)	1.398 (4)	1.395 (4)	1.393 (4)	1.394 (4)
C7—H7	0.95	0.95	0.95	0.95	0.95	0.95
C7—C8	1.402 (4)	1.398 (4)	1.399 (4)	1.396 (4)	1.395 (4)	1.396 (4)
C8—H8	0.95	0.95	0.95	0.95	0.95	0.95
C8—C9	1.383 (5)	1.384 (4)	1.386 (4)	1.388 (4)	1.388 (5)	1.386 (4)
C9—H9	0.95	0.95	0.95	0.95	0.95	0.95
C9—C10	1.395 (4)	1.401 (4)	1.396 (4)	1.393 (4)	1.396 (4)	1.394 (4)
C10—H10	0.95	0.95	0.95	0.95	0.95	0.95
C10—C11	1.390 (4)	1.388 (4)	1.395 (4)	1.396 (4)	1.396 (4)	1.392 (4)
C11—H11	0.95	0.95	0.95	0.95	0.95	0.95
O5—H5C	0.8715	0.8714	0.8711	0.8721	0.8744	0.8696
O5—H5D	0.8692	0.8730	0.8707	0.8694	0.8736	0.8722
O6—H6A	0.8720	0.8705	0.8726	0.8693	0.8712	0.8707
O6—H6B	0.8723	0.8713	0.8706	0.8738	0.8728	0.8716
O7—H7F	0.8723	0.8707		0.8731	0.8704	
O7—H7G	0.8707	0.8714		0.8702	0.8713	

Table S8 Selected angles for **I**·2H2O(av), **I**·2H2O(NS2) and **I**·2H2O(mod).

	AVG	NS2	MOD_{AVG}	MOD_{MIN}	MOD_{MAX}
C3—O3—H3	106.3 (15)	108.4 (5)	105.7 (10)	104.7 (10)	106.9 (10)
C6—O4—C5	117.95 (11)	118.31 (4)	117.80 (10)	117.63 (10)	117.99 (10)

H1A—N1—H1B	106 (2)	102.9 (8)	104.7 (14)	103.0 (14)	106.5 (13)
H1A—N1—H1C	105.8 (18)	109.8 (8)	105.8 (15)	103.2 (15)	108.4 (15)
H1B—N1—H1C	114 (2)	111.6 (8)	113.7 (14)	111.5 (14)	115.9 (15)
C2—N1—H1A	110.3 (14)	109.8 (5)	112.5 (10)	111.5 (10)	113.5 (10)
C2—N1—H1B	108.2 (14)	110.1 (5)	113.3 (10)	111.2 (10)	115.3 (10)
C2—N1—H1C	112.9 (13)	112.3 (5)	106.6 (10)	106.3 (10)	107.1 (10)
O2—C1—O1	126.09 (14)	125.88 (5)	126.09 (13)	125.92 (13)	126.27 (13)
O1—C1—C2	115.21 (13)	115.29 (5)	115.09 (10)	115.01 (10)	115.18 (11)
O2—C1—C2	118.68 (13)	118.80 (5)	118.79 (11)	118.57 (11)	118.99 (11)
N1—C2—C1	110.05 (12)	109.96 (5)	109.93 (9)	109.83 (9)	110.04 (9)
N1—C2—H2	108.3	107.9 (4)	110.52	110.43	110.6
N1—C2—C3	109.28 (12)	109.24 (5)	109.06 (9)	108.94 (9)	109.15 (9)
C1—C2—H2	108.3	108.4 (4)	106.94	106.68	107.23
C1—C2—C3	112.40 (11)	112.44 (4)	112.49 (9)	112.22 (9)	112.73 (9)
C3—C2—H2	108.3	108.7 (4)	107.86	107.75	107.99
O3—C3—C2	107.99 (11)	107.92 (4)	108.14 (9)	107.96 (9)	108.34 (9)
O3—C3—C4	107.10 (12)	107.41 (5)	107.34 (9)	107.25 (9)	107.41 (9)
O3—C3—C5	108.64 (12)	108.58 (5)	108.64 (10)	108.60 (10)	108.68 (10)
C4—C3—C2	112.60 (12)	112.55 (5)	112.59 (9)	112.47 (9)	112.71 (9)
C5—C3—C2	111.16 (11)	111.18 (4)	110.97 (9)	110.85 (9)	111.09 (9)
C4—C3—C5	109.19 (12)	109.06 (5)	109.03 (9)	108.88 (9)	109.19 (9)
C3—C4—H4A	109.5	109.1 (5)	109.47	109.45	109.5
C3—C4—H4B	109.5	112.9 (5)	109.47	109.45	109.49
C3—C4—H4C	109.5	110.7 (5)	109.47	109.41	109.54
H4A—C4—H4B	109.5	108.4 (8)	109.47	109.46	109.48
H4A—C4—H4C	109.5	108.3 (7)	109.47	109.43	109.52
H4B—C4—H4C	109.5	107.2 (7)	109.47	109.45	109.49
O4—C5—C3	106.51 (11)	106.70 (4)	106.43 (10)	106.28 (10)	106.60 (10)
O4—C5—H5A	110.4	111.1 (4)	109.47	109.46	109.48
O4—C5—H5B	110.4	110.5 (5)	109.47	109.46	109.48
C3—C5—H5A	110.4	110.1 (4)	109.47	109.47	109.48
C3—C5—H5B	110.4	107.3 (5)	109.47	109.45	109.5
H5A—C5—H5B	108.6	111.0 (6)	112.35	112.22	112.5
O4—C6—C7	123.83 (14)	123.72 (5)	123.93 (12)	123.73 (12)	124.12 (12)
O4—C6—C11	115.64 (13)	115.83 (5)	115.34 (11)	115.20 (11)	115.48 (11)
C11—C6—C7	120.50 (15)	120.42 (6)	120.69 (11)	120.59 (11)	120.79 (11)
C6—C7—H7	120.6	120.3 (4)	120.56	120.47	120.64
C8—C7—C6	118.88 (16)	119.14 (6)	118.89 (13)	118.72 (13)	119.06 (13)
C8—C7—H7	120.6	120.5 (4)	120.55	120.47	120.64
C7—C8—H8	119.4	118.8 (5)	119.46	119.41	119.5
C9—C8—C7	121.20 (16)	121.03 (6)	121.08 (14)	120.99 (14)	121.17 (14)
C9—C8—H8	119.4	120.1 (5)	119.46	119.41	119.5
C8—C9—H9	120.4	120.5 (5)	120.3	120.28	120.33
C8—C9—C10	119.27 (15)	119.11 (6)	119.39 (14)	119.34 (14)	119.44 (14)
C10—C9—H9	120.4	120.4 (5)	120.3	120.28	120.33
C9—C10—H10	119.7	120.6 (4)	119.66	119.64	119.67

C11—C10—C9	120.62 (16)	120.64 (6)	120.68 (14)	120.65 (14)	120.71 (14)
C11—C10—H10	119.7	118.8 (4)	119.66	119.65	119.67
C10—C11—C6	119.53 (15)	119.66 (6)	119.26 (13)	119.24 (13)	119.28 (13)
C6—C11—H11	120.2	118.1 (5)	120.37	120.36	120.38
C10—C11—H11	120.2	122.2 (5)	120.37	120.36	120.38
H5C—O5—H5D	102 (2)	107.3 (8)	104.2 (11)	102.1 (11)	105.9 (11)
H6A—O6—H6B	104.4 (9)	104.6 (6)	105 (2)	104 (2)	105 (2)
H7A—O7—H7B	104.5 (9)	104.4 (6)	104 (7)	104 (7)	105 (6)

Table S9 Selected angles for **I**·2H2O(supercell).

	No Suffix	A	B	C	D	E
C3—O3—H3	110	110	110	110	110	109.50
C6—O4—C5	118.06 (19)	117.9 (2)	118.1 (2)	118.1 (2)	118.2 (2)	118.02 (19)
H1A—N1—H1B	110	110	110	110	110	109.50
H1A—N1—H1C	110	110	110	110	110	109.50
H1B—N1—H1C	110	110	110	110	110	109.50
C2—N1—H1A	110	110	110	110	110	109.50
C2—N1—H1B	110	110	110	110	110	109.50
C2—N1—H1C	110	110	110	110	110	109.50
O1—C1—C2	115.2 (2)	115.2 (2)	115.2 (2)	115.3 (2)	126.1 (2)	126.0 (2)
O2—C1—O1	125.9 (2)	126.1 (2)	125.9 (2)	125.7 (2)	115.2 (2)	115.3 (2)
O2—C1—C2	118.9 (2)	118.6 (2)	118.9 (2)	118.9 (2)	118.8 (2)	118.6 (2)
N1—C2—C1	109.92 (19)	110.1 (2)	109.81 (19)	110.14 (19)	109.9 (2)	110.16 (19)
N1—C2—H2	108	108	109	109	109	108.40
N1—C2—C3	109.18 (19)	109.2 (2)	109.28 (19)	108.80 (19)	109.2 (2)	108.88 (19)
C1—C2—H2	108	108	109	109	109	108.40
C1—C2—C3	112.58 (19)	112.5 (2)	112.15 (19)	112.13 (19)	112.0 (2)	112.51 (19)
C3—C2—H2	108	108	109	109	109	108.40
O3—C3—C2	107.94 (18)	107.97 (19)	108.17 (18)	107.85 (18)	107.9 (2)	107.87 (18)
O3—C3—C4	107.0 (2)	107.1 (2)	107.19 (19)	107.5 (2)	107.7 (2)	107.5 (2)
O3—C3—C5	108.6 (2)	108.7 (2)	108.69 (19)	108.59 (19)	108.4 (2)	108.42 (19)
C4—C3—C2	112.8 (2)	112.7 (2)	112.67 (19)	112.9 (2)	112.6 (2)	112.89 (19)
C5—C3—C2	111.12 (19)	109.3 (2)	109.02 (19)	109.1 (2)	109.6 (2)	109.42 (19)
C5—C3—C4	109.2 (2)	111.0 (2)	110.96 (19)	110.79 (19)	110.5 (2)	110.55 (19)
C3—C4—H4A	110	110	110	110	110	109.50

C3—C4—H4B	110	110	110	110	110	109.50
C3—C4—H4C	110	110	110	110	110	109.50
H4A—C4—H4B	110	110	110	110	110	109.50
H4A—C4—H4C	110	110	110	110	110	109.50
H4B—C4—H4C	110	110	110	110	110	109.50
O4—C5—C3	106.69 (19)	106.6 (2)	106.43 (19)	106.64 (19)	106.8 (2)	107.03 (18)
O4—C5—H5A	110	110	110	110	110	110.30
O4—C5—H5B	110	110	110	110	110	110.30
C3—C5—H5A	110	110	110	110	110	110.30
C3—C5—H5B	110	110	110	110	110	110.30
H5A—C5—H5B	109	109	109	109	109	108.60
O4—C6—C7	123.4 (2)	123.5 (2)	123.8 (2)	123.7 (2)	123.6 (2)	123.5 (2)
O4—C6—C11	115.5 (2)	115.6 (2)	115.4 (2)	115.3 (2)	115.6 (2)	115.6 (2)
C11—C6—C7	121.1 (2)	120.8 (2)	120.8 (2)	120.9 (2)	120.7 (3)	120.8 (2)
C6—C7—H7	121	121	121	121	121	120.60
C6—C7—C8	118.4 (3)	118.6 (3)	118.6 (3)	118.6 (3)	118.8 (3)	118.7 (3)
C8—C7—H7	121	121	121	121	121	120.60
C7—C8—H8	120	119	119	119	119	119.40
C9—C8—C7	121.1 (3)	121.3 (3)	121.3 (3)	121.2 (3)	121.1 (3)	121.1 (3)
C9—C8—H8	120	119	119	119	119	119.40
C8—C9—H9	120	120	120	120	120	120.30
C8—C9—C10	119.5 (3)	119.4 (3)	119.5 (3)	119.5 (3)	119.6 (3)	119.5 (3)
C10—C9—H9	120	120	120	120	120	120.30
C9—C10—H10	120	120	120	120	120	119.80
C11—C10—C9	120.5 (3)	120.3 (3)	120.3 (3)	120.4 (3)	120.1 (3)	120.4 (3)
C11—C10—H10	120	120	120	120	120	119.80
C6—C11—H11	120	120	120	119.3 (3)	119.7 (3)	120.20
C10—C11—C6	119.3 (3)	119.6 (3)	119.5 (3)	120	120	119.5 (3)
C10—C11—H11	120	120	120	120	120	120.20
H5C—O5—H5D	109.9	104.6	104.8	109.9	104.2	104.8
H6A—O6—H6B	104.4	104.4	104.4	104.3	104.4	109.3
H7F—O7—H7G	104.6	104.4		104.6	104.4	

Table S10 Selected torsion angles for **I**·2H2O(av), **I**·2H2O(NS2) and **I**·2H2O(mod).

	AVG	NS2	MOD$_{AVG}$	MOD$_{MIN}$	MOD$_{MAX}$
O1—C1—C2—N1	176.09(12)	176.28 (5)	176.27 (9)	175.91 (9)	176.63 (9)
O1—C1—C2—C3	−61.86 (16)	−61.76 (5)	-61.99 (12)	-62.41 (12)	-61.57 (12)
O2—C1—C2—N1	−5.51 (18)	−5.44 (6)	-5.59 (14)	-6.00 (14)	-5.18 (14)

O2—C1—C2—C3	116.54 (14)	116.53 (5)	116.15 (11)	115.56 (11)	116.75 (11)
O3—C3—C5—O4	−53.94 (14)	−53.83 (5)	-53.93 (11)	-54.76 (11)	-53.11 (11)
O4—C6—C7—C8	177.07 (15)	177.19 (6)	177.16 (12)	176.58 (12)	177.74 (12)
O4—C6—C11—C10	−176.95 (14)	−177.00 (5)	-176.93 (11)	-177.57 (11)	-176.29 (11)
N1—C2—C3—O3	−41.70 (15)	−41.98 (5)	-41.85 (11)	-43.40 (11)	-40.32 (11)
N1—C2—C3—C4	76.34 (15)	76.35 (5)	76.57 (11)	74.87 (11)	78.26 (11)
N1—C2—C3—C5	−160.78 (12)	−160.96 (5)	-160.91 (9)	-162.36 (10)	-159.46 (10)
C1—C2—C3—O3	−164.19 (12)	−164.36 (4)	-164.09 (9)	-165.90 (8)	-162.28 (9)
C1—C2—C3—C4	−46.15 (16)	−46.02 (6)	-45.67 (12)	-47.64 (12)	-43.70 (12)
C1—C2—C3—C5	76.73 (15)	76.67 (5)	76.85 (11)	75.14 (12)	78.57 (11)
C2—C3—C5—O4	64.76 (14)	64.75 (5)	64.83 (12)	63.76 (12)	65.89 (12)
C4—C3—C5—O4	−170.42 (12)	−170.57 (5)	-170.60 (9)	-171.44 (9)	-169.76 (9)
C5—O4—C6—C7	15.1 (2)	15.20(8)	15.18 (17)	13.15 (17)	17.21 (17)
C5—O4—C6—C11	−166.93 (13)	-166.89(5)	-167.03 (11)	-169.23 (11)	-164.84 (11)
C6—O4—C5—C3	175.06 (12)	175.00(5)	175.01 (10)	173.67 (10)	176.37 (10)
C6—C7—C8—C9	0.3 (3)	0.13 (7)	0.01 (14)	-0.1 (2)	0.1 (2)
C7—C6—C11—C10	1.1 (2)	0.99(9)	0.93 (19)	0.34 (19)	1.52 (19)
C7—C8—C9—C10	−0.1 (3)	0.03 (8)	0.1 (2)	-0.3 (2)	0.4 (2)
C8—C9—C10—C11	0.4 (3)	0.33 (8)	0.3 (2)	0.0 (2)	0.6 (2)
C9—C10—C11—C6	−0.9 (2)	−0.83 (7)	-0.8 (2)	-1.1 (2)	-0.6 (2)
C11—C6—C7—C8	−0.8 (2)	-0.64(9)	-0.51 (17)	-1.01 (19)	0.00 (18)

Table S11 Selected torsion angles for **I**·2H2O(supercell).

	No Suffix	A	B	C	D	E
O1—C1—C2—N1	176.5 (2)	176.0 (2)	176.0 (2)	176.6 (2)	175.7 (2)	176.3 (2)
O1—C1—C2—C3	−61.6 (3)	−62.0 (3)	−62.3 (3)	−62.1 (3)	−62.7 (3)	−62.0 (3)
O2—C1—C2—N1	−5.4 (3)	−5.7 (3)	−6.1 (3)	−5.4 (3)	−5.8 (3)	−5.2 (3)
O2—C1—C2—C3	116.6 (2)	116.4 (3)	115.6 (2)	115.9 (2)	115.8 (3)	116.5 (2)
O3—C3—C5—O4	−54.0 (2)	−54.3 (2)	−53.0 (2)	−53.1 (2)	−53.3 (3)	−54.2 (2)
O4—C6—C7—C8	177.4 (3)	177.7 (2)	176.7 (3)	176.6 (2)	177.2 (3)	177.2 (3)
O4—C6—C11—C10	−177.1 (3)	−177.7 (2)	−176.7 (2)	−176.6 (2)	−177.3 (2)	−177.4 (3)
N1—C2—C3—O3	−42.7 (3)	−42.1 (3)	−40.6 (2)	−41.8 (2)	−41.2 (3)	−43.4 (2)
N1—C2—C3—C4	75.3 (2)	76.0 (3)	77.7 (2)	76.8 (2)	77.4 (3)	75.2 (2)
N1—C2—C3—C5	−161.72 (19)	−161.1 (2)	−159.70 (19)	−160.50 (19)	−159.6 (2)	−161.81 (19)
C1—C2—C3—O3	−165.06 (19)	−164.7 (2)	−162.57 (19)	−163.85 (19)	−163.2 (2)	−165.83 (19)
C1—C2—C3—C4	−47.1 (3)	−46.6 (3)	−44.3 (3)	−45.3 (3)	−44.6 (3)	−47.2 (3)
C1—C2—C3—C5	75.9 (2)	76.3 (3)	78.3 (2)	77.4 (2)	78.4 (3)	75.8 (2)
C2—C3—C5—O4	64.6 (2)	64.3 (3)	65.8 (2)	65.2 (2)	64.8 (3)	63.9 (2)
C4—C3—C5—O4	−170.4 (2)	−170.9 (2)	−169.56 (19)	−169.9 (2)	−170.5 (2)	−171.2 (2)
C5—O4—C6—C7	15.7 (4)	15.7 (3)	13.1 (4)	14.2 (4)	13.9 (4)	16.6 (4)
C5—O4—C6—C11	−166.1 (2)	−166.1 (2)	−169.2 (2)	−168.0 (2)	−168.1 (2)	−165.3 (2)
C6—O4—C5—C3	174.3 (2)	174.8 (2)	176.2 (2)	175.3 (2)	175.9 (2)	173.9 (2)
C6—C7—C8—C9	−0.1 (4)	0.3 (4)	0.3 (4)	0.5 (4)	0.5 (4)	0.9 (4)
C7—C6—C11—C10	1.2 (4)	0.6 (4)	1.1 (4)	1.4 (4)	0.8 (4)	0.7 (4)

C7—C8—C9—C10	0.5 (5)	−0.4 (4)	−0.1 (5)	0.1 (4)	−0.4 (5)	−0.8 (5)
C8—C9—C10—C11	0.0 (5)	0.5 (4)	0.3 (5)	0.1 (4)	0.6 (5)	0.7 (5)
C9—C10—C11—C6	−0.9 (4)	−0.6 (4)	−0.8 (4)	−0.8 (4)	−0.7 (4)	−0.6 (4)
C11—C6—C7—C8	−0.7 (4)	−0.4 (4)	−0.9 (4)	−1.2 (4)	−0.7 (4)	−0.8 (4)

S4. Refinement Parameters for I·2H2O(mod)

Table S12 Occupational harmonic wave parameters for molecules containing O6 and O7 in **I**·2H2O(mod).

Molecule			
Water6		Occupancy	0.624(3)
s,1	0.011(2)	s,2	-0.013(13)
c,1	0.498(3)	c,2	-0.117(8)
Water7		Occupancy	0.376(3)
s,1	-0.046(2)	s,2	0.029(13)
c,1	-0.496(3)	c,2	0.114(8)

Table S13 Positional and harmonic displacive parameters for molecules containing O6 and O7 in **I**·2H2O(mod).

Molecule		x-trans	y-trans	z-trans	x-rot	y-rot	z-rot
Water6		0	0	0	0.0027(5)	-0.0023(3)	0.00001(5)
	s,1	-0.009(3)	0.005(3)	0.0013(5)	0.0003(2)	-0.00826(18)	0.00278(4)
	c,1	0.014(6)	0.004(5)	-0.0030(13)	-0.0055(7)	0.0047(4)	0.00004(8)
Water7		0	0	0	-0.015(3)	0.0110(14)	-0.0007(3)
	s,1	0.004(6)	-0.008(6)	-0.0001(13)	-0.0014(9)	-0.0026(5)	0.00111(8)
	c,1	-0.014(9)	0.000(13)	-0.002(3)	-0.025(3)	0.0157(17)	-0.0012(3)

Table S14 Average atomic position parameters for all atoms in the molecules containing O6 and O7 in **I**·2H2O(mod).

Atom	x	y	z	Ueq/Uiso
O6	1.0229	0.7634	0.4807	0.0180(3)
H6a	1.173(2)	0.740(5)	0.4691(6)	0.026976
H6b	1.053(4)	0.756(5)	0.50731(13)	0.026976
O7	0.9075	0.7816	0.4844	0.0257(5)
H7a	0.765(5)	0.848(11)	0.4761(16)	0.038612
H7b	0.888(10)	0.765(8)	0.5111(3)	0.038612

Table S15 Average harmonic ADPs for atoms O6 and O7 in **I**·2H2O(mod).

Atom	U11	U22	U33	U12	U13	U23
O6	0.0130(6)	0.0234(4)	0.0175(4)	-0.0014(3)	0.0000(3)	-0.0045(3)
O7	0.0226(12)	0.0314(7)	0.0232(6)	0.0032(6)	-0.0010(5)	-0.0088(5)

Table S16 Positional and harmonic displacive parameters for all atoms in the zwitterion and the water molecule containing O5 in **I**·2H2O(mod).

Atom	Occ.	Wave	x	y	z	Ueq/Uiso
O1	1		0.77486(11)	0.35341(10)	0.589910(18)	0.01462(16)
		s,1	-0.00834(12)	0.00169(10)	0.002732(18)	
		c,1	0.00209(12)	-0.00150(10)	0.000177(18)	
O2	1		0.75255(11)	0.17812(10)	0.534833(18)	0.01484(16)
		s,1	-0.00282(13)	-0.00688(10)	0.003360(18)	
		c,1	0.00236(12)	0.00155(10)	0.000955(18)	
O3	1		0.09676(11)	0.04131(10)	0.605155(16)	0.01295(16)
		s,1	-0.00755(12)	0.00378(10)	0.003210(18)	
		c,1	-0.00438(13)	0.00031(10)	0.001224(19)	
H3	1		0.018(2)	0.1550(14)	0.6051(4)	0.019428
		s,1	-0.005(2)	0.0029(15)	0.0013(3)	
		c,1	0.001(2)	0.0011(15)	0.0010(3)	
O4	1		0.20678(12)	0.40123(10)	0.641673(17)	0.01535(17)
		s,1	-0.00107(12)	0.00790(10)	0.001504(17)	
		c,1	-0.00059(13)	-0.00764(10)	0.000096(17)	
N1	1		0.28382(13)	0.13325(12)	0.537229(19)	0.01340(18)
		s,1	-0.00220(15)	-0.01277(13)	0.00284(2)	
		c,1	0.00216(15)	-0.00223(13)	0.00066(2)	
H1a	1		0.301(2)	0.2103(18)	0.5158(2)	0.016082
		s,1	-0.001(2)	-0.0150(16)	0.0027(3)	
		c,1	-0.001(2)	0.0016(16)	0.0001(3)	
H1b	1		0.346(2)	0.0042(13)	0.5315(3)	0.016082
		s,1	-0.003(2)	-0.0091(14)	0.0026(3)	
		c,1	0.003(2)	-0.0030(14)	0.0011(3)	
H1c	1		0.1276(12)	0.130(2)	0.5419(3)	0.016082
		s,1	-0.0031(15)	-0.0078(17)	0.0021(3)	
		c,1	0.0017(15)	-0.0033(17)	0.0003(3)	
C1	1		0.66400(15)	0.25824(12)	0.56411(2)	0.0113(2)
		s,1	-0.00511(18)	-0.00336(13)	0.00298(3)	
		c,1	0.00118(15)	0.00078(13)	0.00040(3)	
C2	1		0.39707(15)	0.23551(12)	0.57080(2)	0.0099(2)
		s,1	-0.00546(17)	-0.00561(13)	0.00254(2)	
		c,1	0.00096(15)	-0.00029(13)	0.00023(2)	
H2	1		0.333	0.3813	0.5746	0.011921
		s,1	-0.0068	-0.0053	0.0016	
		c,1	0.003	0.0013	-0.0002	
C3	1		0.33896(14)	0.10323(13)	0.60703(2)	0.0105(2)
		s,1	-0.00689(16)	0.00138(13)	0.00308(2)	
		c,1	-0.00270(16)	-0.00183(13)	0.00096(2)	
C4	1		0.48580(15)	-0.09801(13)	0.60976(2)	0.0153(2)
		s,1	-0.00703(17)	0.00114(14)	0.00332(3)	
		c,1	-0.00871(17)	-0.00230(14)	0.00270(3)	
H4a	1		0.4258	-0.1861	0.6304	0.022982
		s,1	-0.005	0.0051	0.0032	
		c,1	-0.0059	0.0046	0.0045	

H4b	1		0.4751	-0.1761	0.5859	0.022982
		s,1	-0.0072	-0.0023	0.0032	
		c,1	-0.0182	-0.0083	0.004	
H4c	1		0.6509	-0.0613	0.6147	0.022982
		s,1	-0.0066	0.0003	0.0027	
		c,1	-0.0061	-0.0037	0.0011	
C5	1		0.37622(15)	0.23453(13)	0.64294(2)	0.0123(2)
		s,1	-0.00594(18)	0.00610(13)	0.00251(2)	
		c,1	-0.00100(16)	-0.00457(13)	0.00031(2)	
H5a	1		0.3468	0.1453	0.6655	0.014809
		s,1	-0.0094	0.0117	0.0029	
		c,1	-0.0032	-0.0038	0.0007	
H5b	1		0.5374	0.2952	0.6428	0.014809
		s,1	-0.0041	0.0014	0.0025	
		c,1	-0.0004	-0.0049	0.0002	
C6	1		0.19156(15)	0.53282(13)	0.67244(2)	0.0129(2)
		s,1	-0.00249(17)	0.00583(14)	0.00143(2)	
		c,1	-0.00095(18)	-0.01335(14)	0.00014(3)	
C7	1		0.36283(16)	0.54550(14)	0.70072(2)	0.0159(2)
		s,1	-0.00327(18)	0.00528(15)	0.00156(3)	
		c,1	-0.00153(18)	-0.01659(15)	0.00028(3)	
H7	1		0.4973	0.4557	0.7002	0.019114
		s,1	-0.0027	0.0087	0.0016	
		c,1	-0.0027	-0.0158	0.0005	
C8	1		0.33347(18)	0.69234(16)	0.72989(3)	0.0210(2)
		s,1	-0.0050(2)	-0.00096(16)	0.00165(3)	
		c,1	0.0000(2)	-0.01939(17)	0.00007(3)	
H8	1		0.4493	0.7021	0.7493	0.025227
		s,1	-0.0057	-0.0019	0.0018	
		c,1	-0.0002	-0.0213	0.0001	
C9	1		0.13815(18)	0.82407(16)	0.73102(3)	0.0224(3)
		s,1	-0.0046(2)	-0.00630(16)	0.00160(3)	
		c,1	0.0019(2)	-0.01715(16)	-0.00018(3)	
H9	1		0.1202	0.9235	0.751	0.026826
		s,1	-0.0044	-0.0108	0.0017	
		c,1	0.0039	-0.0158	-0.0004	
C10	1		-0.03148(18)	0.80945(15)	0.70263(3)	0.0201(2)
		s,1	-0.0036(2)	-0.00455(16)	0.00144(3)	
		c,1	0.00136(19)	-0.01552(15)	-0.00006(3)	
H10	1		-0.1653	0.9	0.7032	0.024077
		s,1	-0.0031	-0.0075	0.0014	
		c,1	0.0022	-0.0142	-0.0001	
C11	1		-0.00726(16)	0.66341(13)	0.67335(3)	0.0165(2)
		s,1	-0.00283(18)	0.00129(15)	0.00135(3)	
		c,1	-0.00018(18)	-0.01457(15)	0.00006(3)	
H11	1		-0.1247	0.6527	0.6542	0.019747

		s,1	-0.0023	0.0017	0.0013	
		c,1	-0.0005	-0.0134	0.0001	
O5	1		0.98748(13)	0.69494(11)	0.559084(18)	0.02150(18)
		s,1	0.00187(15)	-0.00315(11)	0.00245(2)	
		c,1	-0.01957(15)	0.01613(12)	-0.00021(2)	
H5c	1		0.931(3)	0.5734(12)	0.5719(3)	0.032254
		s,1	-0.004(2)	-0.0012(11)	0.0029(3)	
		c,1	0.012(2)	0.0035(12)	0.0004(3)	
H5d	1		1.016(3)	0.7963(14)	0.5786(2)	0.032254
		s,1	-0.006(3)	0.0048(13)	0.0017(2)	
		c,1	-0.004(2)	0.0053(13)	0.0005(2)	

Table S17 Average harmonic ADPs and 2nd ADP modulation parameters for the zwitterion and the water molecule containing O5 in **I**·2H2O(mod).

Atom	Wave	U11	U22	U33	U12	U13	U23
O1		0.0111(3)	0.0142(3)	0.0186(3)	-0.0031(2)	-0.0028(2)	0.0001(2)
	s,1	-0.0003(3)	-0.0006(3)	-0.0018(3)	0.0005(2)	0.0006(3)	-0.0011(2)
	c,1	-0.0011(3)	-0.0007(3)	-0.0044(3)	0.0000(2)	0.0017(3)	0.0005(2)
O2		0.0105(3)	0.0181(3)	0.0159(3)	0.0008(2)	0.0019(2)	0.0011(2)
	s,1	0.0007(3)	0.0015(3)	-0.0003(3)	0.0006(3)	-0.0007(2)	-0.0011(2)
	c,1	-0.0007(3)	-0.0011(3)	-0.0075(3)	0.0003(2)	0.0001(2)	0.0029(2)
O3		0.0084(3)	0.0128(3)	0.0176(3)	-0.0019(2)	-0.0004(2)	0.0014(2)
	s,1	-0.0004(3)	-0.0002(3)	0.0031(3)	0.0002(3)	-0.0002(2)	-0.0002(2)
	c,1	-0.0010(3)	-0.0002(3)	-0.0059(3)	0.0003(2)	0.0019(2)	-0.0008(2)
O4		0.0155(3)	0.0178(3)	0.0128(3)	0.0052(2)	-0.0029(2)	-0.0033(2)
	s,1	-0.0002(3)	-0.0021(3)	-0.0012(3)	0.0003(3)	-0.0003(2)	0.0004(2)
	c,1	0.0004(4)	0.0000(3)	-0.0011(3)	-0.0001(3)	-0.0001(2)	-0.0009(2)
N1		0.0099(3)	0.0191(3)	0.0112(3)	-0.0006(3)	-0.0008(2)	-0.0015(2)
	s,1	0.0001(4)	-0.0021(4)	0.0020(4)	0.0014(3)	-0.0003(3)	-0.0014(3)
	c,1	-0.0005(4)	-0.0044(4)	-0.0050(4)	0.0003(3)	-0.0002(3)	0.0038(3)
C1		0.0095(4)	0.0093(4)	0.0152(4)	-0.0002(3)	-0.0002(3)	0.0030(3)
	s,1	-0.0008(5)	0.0007(4)	-0.0005(4)	0.0003(3)	0.0003(3)	-0.0002(3)
	c,1	0.0000(4)	-0.0007(4)	-0.0052(4)	-0.0004(3)	0.0009(3)	0.0015(3)
C2		0.0084(4)	0.0104(3)	0.0110(3)	0.0002(3)	-0.0015(3)	0.0000(3)
	s,1	-0.0005(5)	-0.0006(4)	-0.0001(4)	0.0003(3)	-0.0002(3)	-0.0002(3)
	c,1	-0.0001(4)	-0.0013(4)	-0.0036(4)	-0.0005(3)	0.0006(3)	0.0020(3)
C3		0.0089(4)	0.0105(3)	0.0120(3)	-0.0009(3)	-0.0008(3)	0.0007(3)
	s,1	0.0014(5)	-0.0003(4)	0.0014(4)	0.0003(3)	-0.0009(3)	-0.0006(3)
	c,1	-0.0015(4)	0.0005(4)	-0.0056(4)	0.0005(3)	0.0016(3)	0.0003(3)
C4		0.0132(4)	0.0116(4)	0.0211(3)	0.0011(3)	-0.0019(3)	0.0031(3)
	s,1	0.0007(5)	0.0006(4)	0.0048(5)	0.0006(3)	-0.0020(4)	-0.0005(3)
	c,1	-0.0024(5)	-0.0001(4)	-0.0075(5)	-0.0008(4)	0.0038(4)	0.0010(3)
C5		0.0108(4)	0.0142(4)	0.0120(3)	0.0009(3)	-0.0017(3)	0.0002(3)
	s,1	-0.0007(5)	-0.0013(4)	-0.0001(4)	0.0006(3)	0.0002(3)	-0.0001(3)

	c,1	0.0000(4)	-0.0002(4)	-0.0042(4)	-0.0001(3)	0.0012(3)	-0.0010(3)
C6		0.0145(4)	0.0132(4)	0.0110(3)	-0.0028(3)	0.0018(3)	0.0006(3)
	s,1	-0.0002(5)	-0.0008(4)	-0.0012(4)	0.0004(3)	-0.0010(3)	-0.0007(3)
	c,1	-0.0005(5)	-0.0001(4)	-0.0004(4)	-0.0001(3)	-0.0003(3)	-0.0015(3)
C7		0.0159(4)	0.0187(4)	0.0132(4)	0.0000(3)	-0.0010(3)	-0.0005(3)
	s,1	0.0002(5)	-0.0011(4)	-0.0003(4)	0.0002(4)	-0.0005(3)	-0.0017(3)
	c,1	-0.0004(5)	-0.0005(5)	-0.0007(4)	-0.0004(4)	-0.0002(4)	0.0000(3)
C8		0.0228(5)	0.0248(4)	0.0154(4)	-0.0033(4)	-0.0031(3)	-0.0035(3)
	s,1	-0.0001(6)	0.0008(5)	-0.0001(4)	0.0004(4)	-0.0010(4)	-0.0018(4)
	c,1	-0.0012(6)	-0.0019(6)	0.0001(5)	-0.0006(4)	-0.0003(4)	0.0006(4)
C9		0.0286(5)	0.0194(4)	0.0190(4)	-0.0020(4)	0.0030(4)	-0.0065(3)
	s,1	0.0010(6)	0.0004(5)	-0.0008(5)	0.0005(4)	-0.0005(4)	-0.0019(4)
	c,1	-0.0008(6)	-0.0023(5)	0.0003(5)	-0.0011(4)	-0.0006(4)	0.0003(4)
C10		0.0214(4)	0.0184(4)	0.0204(4)	0.0020(3)	0.0050(3)	-0.0016(3)
	s,1	0.0005(6)	0.0000(5)	-0.0008(4)	0.0003(4)	0.0000(4)	-0.0017(4)
	c,1	-0.0007(6)	-0.0021(5)	-0.0002(5)	-0.0019(4)	-0.0008(4)	0.0000(4)
C11		0.0167(4)	0.0175(4)	0.0151(4)	0.0006(3)	0.0002(3)	0.0002(3)
	s,1	0.0006(5)	-0.0009(4)	-0.0006(4)	0.0005(4)	-0.0001(4)	-0.0014(3)
	c,1	0.0003(5)	-0.0004(5)	-0.0002(4)	-0.0014(4)	0.0002(4)	-0.0002(3)
O5		0.0255(4)	0.0197(3)	0.0193(3)	-0.0088(3)	0.0026(2)	-0.0023(2)
	s,1	0.0000(5)	-0.0017(3)	-0.0009(3)	0.0005(3)	-0.0041(3)	0.0049(3)
	c,1	-0.0069(4)	-0.0089(4)	-0.0019(3)	0.0074(3)	-0.0036(3)	0.0026(3)
